# Reservoir dynamics of rabies in south-east Tanzania and the roles of cross-species transmission and domestic dog vaccination

**DOI:** 10.1111/1365-2664.13983

**Published:** 2021-11

**Authors:** Kennedy Lushasi, Sarah Hayes, Elaine A. Ferguson, Joel Changalucha, Sarah Cleaveland, Nicodem J. Govella, Daniel T. Haydon, Maganga Sambo, Geofrey J. Mchau, Emmanuel A. Mpolya, Zacharia Mtema, Hezron E. Nonga, Rachel Steenson, Pierre Nouvellet, Christl A. Donnelly, Katie Hampson

**Affiliations:** 1 Ifakara Health Institute, Ifakara, Tanzania; 2 Department of Infectious Disease Epidemiology, Faculty of Medicine, School of Public Health, Imperial College London; 3 Institute of Biodiversity, Animal Health and Comparative Medicine, University of Glasgow, Glasgow, UK; 4 Nelson Mandela African Institution of Science and Technology, Arusha, Tanzania; 5 Ministry of Health, Community Development, Gender, Elderly and Children, Dodoma, Tanzania; 6 Ministry of Livestock Development and Fisheries, Dodoma, Tanzania; 7 School of Life Sciences, University of Sussex, Brighton, UK; 8 Department of Statistics, University of Oxford, Oxford, UK

**Keywords:** dog-mediated rabies, lyssavirus, One Health, spillover, surveillance, vaccination, zero by thirty, zoonoses

## Abstract

1. Understanding the role of different species in the transmission of multi-host pathogens, such as rabies virus, is vital for effective control strategies. Across most of sub-Saharan Africa domestic dogs *Canis familiaris* are considered the reservoir for rabies, but the role of wildlife has been long debated. Here we explore the multi-host transmission dynamics of rabies across south-east Tanzania.
2. Between January 2011 and July 2019, data on probable rabies cases were collected in the regions of Lindi and Mtwara. Hospital records of animal-bite patients presenting to healthcare facilities were used as sentinels for animal contact tracing. The timing, location and species of probable rabid animals were used to reconstruct transmission trees to infer who infected whom and the relative frequencies of within- and between-species transmission.
3. During the study, 688 probable human rabies exposures were identified, resulting in 47 deaths. Of these exposures, 389 were from domestic dogs (56.5%) and 262 from jackals (38.1%). Over the same period, 549 probable animal rabies cases were traced: 303 in domestic dogs (55.2%) and 221 in jackals (40.3%), with the remainder in domestic cats and other wildlife species.
4. Although dog-to-dog transmission was most commonly inferred (40.5% of transmission events), a third of inferred events involved wildlife-to-wildlife transmission (32.6%), and evidence suggested some sustained transmission chains within jackal populations.
5. A steady decline in probable rabies cases in both humans and animals coincided with the implementation of widespread domestic dog vaccination during the first 6 years of the study. Following the lapse of this program, dog rabies cases began to increase in one of the northernmost districts.
6. *Synthesis and applications*. In south-east Tanzania, despite a relatively high incidence of rabies in wildlife and evidence of wildlife-to-wildlife transmission, domestic dogs remain essential to the reservoir of infection. Continued dog vaccination alongside improved surveillance would allow a fuller understanding of the role of wildlife in maintaining transmission in this area. Nonetheless, dog vaccination clearly suppressed rabies in both domestic dog and wildlife populations, reducing both public health and conservation risks and, if sustained, has potential to eliminate rabies from this region.

## 1 Introduction

Rabies virus (RABV) is a true multi-host pathogen. Although typically maintained in distinct species-specific transmission cycles (Rupprecht et al., 2002), the virus is capable of infecting any mammal. Rabies is spread primarily through bites from infected animals and cross-species transmission causes disease in humans, livestock and wildlife (Hikufe et al., 2019). Every year an estimated 59,000 human rabies deaths occur (Hampson et al., 2015), mostly in low- and middle-income countries (LMICs; WHO, 2018). The economic burden of rabies due to livestock losses is high, and rabies outbreaks within wildlife can threaten endangered species (Randall et al., 2004).

**Figure 1:**
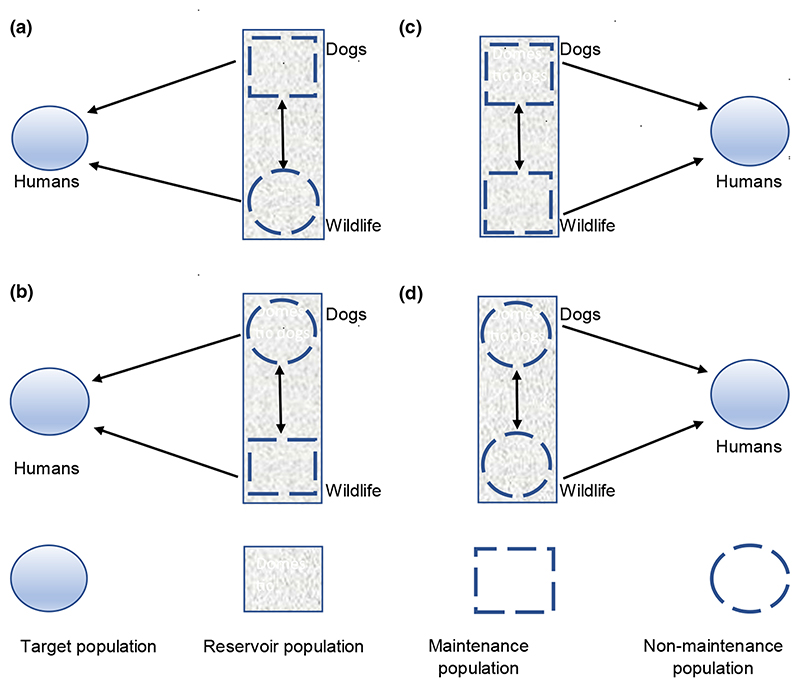
Potential rabies reservoir systems in south-east Tanzania. Here humans are indicated as the target population, but the target may include livestock or endangered wildlife, for example African Wild Dogs (*Lycaon pictus*). We investigate whether the reservoir consists of both maintenance and non-maintenance populations (a and b) transmitting infection to the non-maintenance target (humans); or either two maintenance (c) or non-maintenance (d) populations which are capable of transmitting infection to the target

When planning control and elimination strategies for multi-host pathogens, it is important to identify the populations that are essential for their persistence (Haydon et al., 2002; Roberts & Heesterbeek, 2003). Throughout this study, we use terminology defined by Haydon et al. (2002) and illustrated in Figure 1. That is, a single population capable of independently maintaining the pathogen of interest is termed a maintenance population. Where multiple interconnected host populations collectively maintain the pathogen this is termed a maintenance community. A reservoir is made up of one or more epidemiologically connected populations capable of permanently maintaining the pathogen and from which infection is transmitted to a population of concern (the target population). If a single maintenance population exists, control measures targeted at this population should lead to elimination of infection from all populations. In the presence of a maintenance community, interventions may either need to be targeted at multiple populations or intensified if only one of the host types within a maintenance community is targeted (Roberts & Heesterbeek, 2003).

The global eradication of rinderpest is one example of the success of interventions targeted at a maintenance population. Although capable of infecting more than 40 domestic and wild artiodactyl species, cattle *Bos taurus* vaccination alone eradicated rinderpest virus (Youde, 2013). More recently, the importance of understanding reservoir dynamics has been highlighted by the global Guinea Worm Eradication Programme where the recent discovery of dogs as a potential maintenance host for this parasitic infection has complicated eradication efforts (Molyneux & Sankara, 2017).

Although many RABV variants exist, each variant tends to associate closely with a particular mammalian species which serves as the maintenance host for that variant. Spillover of infection to other species does occur, but sustained transmission outside the maintenance population is uncommon. Interventions targeted at the maintenance population should therefore be effective in controlling that variant. However, there are reported instances of multiple species maintaining a single RABV variant, either separately as distinct maintenance populations or together as a combined maintenance community. On some Caribbean islands, dogs and mongooses (family Herpestidae) maintain the same dog-derived RABV variant and act as a combined maintenance community (Nadin-Davis et al., 2008; Velasco-Villa et al., 2017). In such situations, interventions may need to target both populations to achieve elimination. The presence of multiple maintenance populations can also have implications for disease re-emergence. In north-eastern Mexico, a dog/coyote RABV variant was believed to have been eliminated following widespread dog vaccination. However, the variant continued to circulate in the coyote *Canis latrans* population and was subsequently reintroduced to dogs via dog–coyote contact. Sustained transmission was possible given the waning herd immunity from inadequate vaccination coverage (Velasco-Villa et al., 2017).

Domestic dogs are considered the maintenance hosts for RABV in Africa and Asia, and are responsible for 99% of all human rabies deaths (WHO, 2018). However, the diversity of wild carnivores across Africa has led to ongoing debate regarding a role for wildlife in maintaining rabies in this region. Across parts of South Africa and southern Namibia, independently maintained rabies cycles are reported in the bat-eared fox *Otocyon megalotis* (Hikufe et al., 2019; Sabeta et al., 2007; Thomson & Meredith, 1993). Jackal species frequently represent a large proportion of reported wildlife rabies cases in southern Africa (Moagabo et al., 2009; Pfukenyi et al., 2009), and in parts of Namibia, South Africa and Zimbabwe, black-backed jackals *Canis mesomelas* appear to play a role in maintaining transmission (Bellan et al., 2012; Bingham et al., 1999; Courtin et al., 2000; Hikufe et al., 2019; Zulu et al., 2009).

Evidence from northern Tanzania suggests that domestic dogs are the only species necessary for the maintenance of RABV in this area, although other carnivores contribute to the reservoir as non-maintenance populations (Lembo et al., 2008). In contrast, very little is known about the transmission dynamics of RABV in south-east Tanzania. In 2010, the government of Tanzania began a 5-year rabies elimination demonstration project, which involved provision of free post-exposure prophylaxis to bite victims, mass dog vaccination and improved surveillance across 28 districts (Mpolya et al., 2017). The area was selected to include a large wildlife-protected area to enable examination of hypotheses evaluating the role of wildlife areas as buffers against infection, and/or the potential of wildlife to impede elimination efforts.

‘Zero by Thirty’ is an initiative backed by the United Against Rabies Coalition aiming to achieve zero human deaths world-wide from dog-mediated rabies by 2030 (WHO, FAO & OIE, 2015). Vaccination of domestic dogs and disease surveillance are key components of this initiative. Surveillance needs to include approaches for detecting cases in all species, including wildlife, to assess whether and how wildlife infections impact the effectiveness of dog vaccinations. Here we investigate the transmission dynamics of RABV in 13 districts of south-east Tanzania. We hypothesize that if domestic dogs are the sole maintenance hosts of RABV in this region and if a maintenance community does not exist, then control strategies directed at domestic dogs alone should reduce or eliminate RABV. We further examine the evidence for sustained transmission of RABV in wildlife and whether wildlife present an obstacle to elimination which will be particularly important in informing efforts to achieve the 2030 target.

## 2 Materials and Methods

### 2.1 Study area

**Figure 2:**
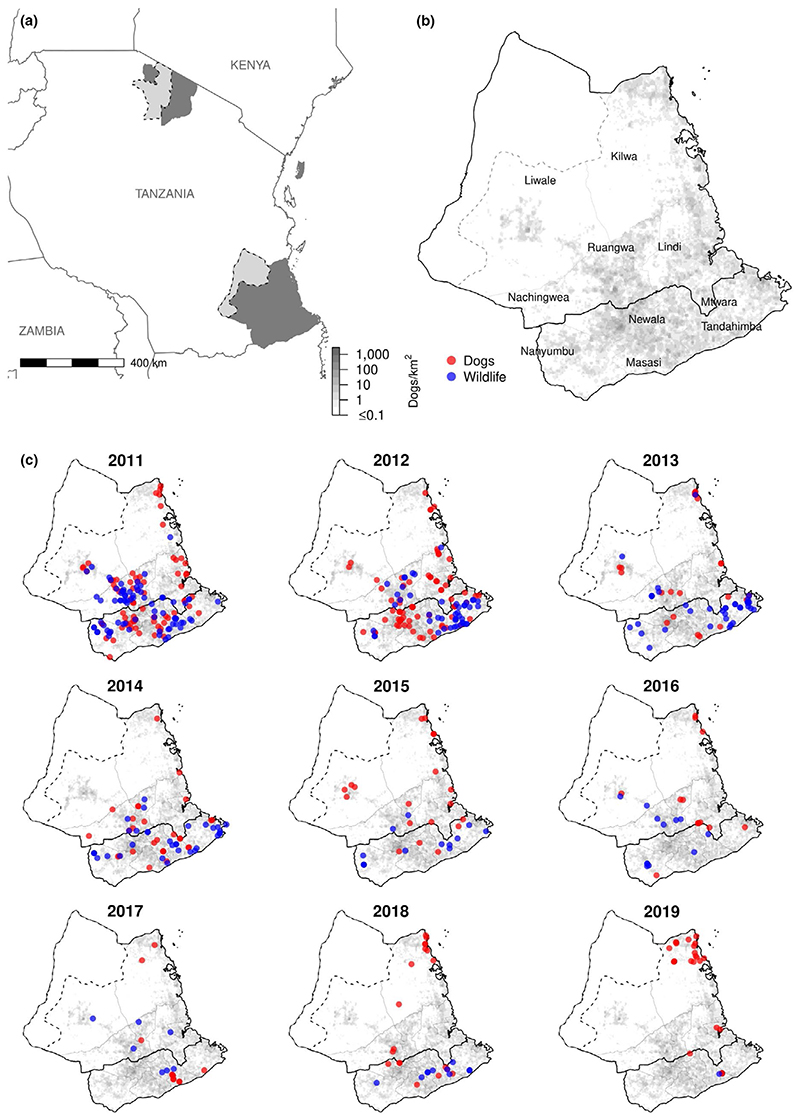
Study districts and locations of probable rabies cases. (a) The study area (dark grey) and protected areas where no human settlements are allowed (light grey): Selous Game Reserve in south-east Tanzania and Serengeti National Park in northern Tanzania. (b) Districts in Lindi and Mtwara regions (labelled) with estimated dog density on a 4-km^2^ raster (grey shading). Urban districts within Masasi, Lindi and Mtwara are not labelled to improve readability. (c) Probable rabies cases in dogs (red) and wildlife (blue) each year in Lindi and Mtwara regions

The study took place between January 2011 and July 2019 within the 13 districts of the Lindi and Mtwara regions of south-east Tanzania, covering an area of 82,668 km^2^ (Figure 2). These districts were part of a World Health Organization (WHO) coordinated demonstration project funded by the Bill and Melinda Gates Foundation to pilot strategies for rabies control and elimination (Mpolya et al., 2017). The study area contains forest reserves, plantations and the wildlife-protected area of the Selous Game Reserve. These areas are potential habitats for wildlife species previously reported to transmit rabies to humans, such as jackals and hyenas *Crocuta crocuta*. The total human population in the study area for 2018 was estimated to be 2,277,552 (United Nation Development Programme, 2018) and the dog population 49,701 extrapolated from post-vaccination transects and human:dog ratios (Sambo et al., 2018). The districts vary dramatically, ranging from <200 to >1,400 km^2^ in area, with human:dog ratios spanning from <35 to >90 persons per dog and dog densities from <0.2 to >7 dogs per km^2^ (Table S1).

Prior to the study, no mass dog vaccinations had been carried out in any of these districts, so dog vaccination coverage was likely negligible. However, with the WHO-coordinated demonstration project, five mass dog vaccination campaigns were implemented in each district between 2010 and late 2016/early 2017 (Mpolya et al., 2017). A central point strategy was used and post-vaccination transects conducted to evaluate the campaigns (further details in Appendix S1). District-level vaccination coverages increased from a median of 23.5% in the first campaign (range across districts: 3.2%–60.1%) to 34.5% in 2014/15 (range: 22.1%–58.0%), with 33.2% coverage (range: 15.8%–54.8%) achieved during the final campaign in late 2016/early 2017 (Table S2). Coverage within districts was highly heterogeneous (Figure S1).

### 2.2 Data collection

A mobile phone-based surveillance system was used across the area to record animal-bite victims presenting to health facilities requiring post-exposure prophylaxis (Mtema et al., 2016). Records from this system were extracted and compared with paper-based records from health facilities, and any additional animal-bite victims from health facilities that were not present in the mobile phone-based surveillance system were identified. All animal-bite victims and owners of biting animals were exhaustively traced and interviewed to obtain details of each bite incident, as described in the study by Hampson et al. (2009). Information collected during interviews included the following: the date and GPS coordinates of the person bitten; if possible, the origin of the biting animal; the species; the dog owner, if known; and whether the animal was known to have bitten other people or animals. Details regarding the animal’s behaviour and the bite circumstances were used to assess whether the animal was considered likely to have been rabid. If additional biting animals or bite victims were identified during investigations, they were also traced and interviewed. Where the GPS location did not correspond to the bite location, a categorical indicator of uncertainty was assigned based on the estimated distance to the biting incident (nearby <2 km away, walking distance 2–5 km and far 5–10 km). We used the resulting data on probable rabies cases and exposures to examine trends in incidence and infer transmission within and between species.

### 2.3 Analyses

#### 2.3.1 Parameter estimation

We define the serial interval as the interval between the onset of clinical signs in a primary case to the onset of clinical signs in a secondary case infected by the primary case. The distance kernel represents the distance between the locations of the primary and secondary cases. The probability distribution of the serial interval and distance kernel for transmission of RABV in domestic dogs were estimated using data on probable rabies cases from a long-term contact tracing study in Serengeti District, northern Tanzania. These data included the date and location of the bite incident for the primary rabid animals and the secondary cases that they infected, with information available for serial interval and distance kernel estimation in 1,139 and 958 cases respectively. The parameters from the Serengeti data were used throughout subsequent analyses, as information regarding the serial interval and distance kernel for known transmission events within the south-east Tanzania data was limited (32 and 15 cases respectively), and thus it was felt that the distributions would be better characterized using the Serengeti data. The best-fitting distributions for data available from south-east Tanzania were estimated and a likelihood ratio test was undertaken to test the null hypothesis that the parameters were the same for the Serengeti and south-east Tanzania. For both parameters, maximum likelihood-based approaches were used for estimation, and the best-fitting parametric form was selected using Akaike’s information criteria (AIC). Details are provided in the Supporting Information (Appendix S2).

#### 2.3.2 Transmission trees

The estimates of the serial interval *G* and distance kernel *K*, described above were used within a previously developed algorithm (Hampson et al., 2009) to generate putative epidemic trees. We define a ‘progenitor’ as a case that was inferred to be the source of infection for another case. For each probable case i, a progenitor j was chosen at random with probability *P_ij_* from all cases within south-east Tanzania with a date of onset of clinical signs prior to the date of onset of the case (*n*), where:

$$p_{ij}=\frac{G\left(t_{ij}\right)K\left(d_{ij}\right)}{\overset{n}{\underset{k=1}{\sum^{\text{​}}}}G\left(t_{ik}\right)K\left(d_{ik}\right)},$$

*t_ij_* is the days between the onset of clinical signs in case *i* and its potential progenitor *j*; and *d_ij_* is the distance between the locations of cases *i* and *j*. For probable cases in wildlife or for dogs where the owner was not known, the convolution of two distance kernels was used to better incorporate the greater uncertainty in reported locations for these cases.

Due to uncertainty around the dates and locations of some cases, 50,000 bootstrapped datasets of plausible progenitors were generated. In each iteration, for cases with uncertainty around the date of onset of clinical signs and/or their location, dates and/or locations were selected randomly from a uniform distribution within the period or radius of uncertainty respectively. For each case, the case selected most frequently as the progenitor within the 50,000 bootstrapped datasets was considered the most likely progenitor.

Cases from all species were included in the analysis. As data to estimate the serial interval and distance kernel for wildlife were lacking, we assumed these distributions for wildlife were the same as those for domestic dogs.

#### 2.3.3 Assessing within- and between-species transmission

The algorithm assigning progenitors does not account for unobserved cases. Attempts to adjust for unobserved cases were made by analysing a subset of inferred transmissions considered most likely to represent direct transmission. Only inferred transmissions below the 99th percentile value of the serial interval and the convolution of two distance kernel distributions were analysed to assess within- and between-species transmission, corresponding to cut-off values of 156 days and 9,803 m respectively. Transmissions with serial intervals and/or distances above these cut-off values were considered less likely to represent direct transmission.

Within this subset of inferred transmissions, relative frequencies of within- and between-species transmission were estimated. Weighted random sampling was used to select a single progenitor for each case from the set of bootstrapped progenitors for that case (selected with replacement from all cases) and the species recorded and used to construct a contingency table of inferred transmissions. Fisher’s exact test statistic was calculated to test whether the inferred levels of inter-species transmission would be expected under random mixing. This procedure was repeated 1,000 times, and median levels of inferred transmission and *p*-values were calculated.

To assess the robustness of the transmission tree results, we conducted sensitivity analyses for different scenarios. These included using an alternative upper limit for the interval censoring of the distance data, using the 95th percentile values of the distributions as the cut-off values, using only the single most likely progenitor in construction of the transmission trees rather than considering all possible progenitors, alternative approaches to addressing the uncertainty in the dates reported and subsampling dog rabies cases to assess how case detection affects inference of within- and between-species transmission. Full details are found in Appendix S3.

#### 2.3.4 Chains of transmission and cluster size

Using the most likely progenitor identified for each case (highest bootstrap support), chains of transmission were constructed and examined for evidence of sustained transmission among domestic animals and/or wildlife. Clusters of cases linked by directly inferred transmissions (see above) were identified, and the sizes of clusters consisting of a single species or mixture of species were evaluated. The mean cluster size (including clusters of one) per 6-month period from the first case recorded was calculated and a weighted linear spline regression performed to test for a temporal trend. A 6-month period was selected to allow full use of the data while allowing a long enough time window for clusters to be observed. Sensitivity analyses were performed using periods of 3 months and 1 year, with the 2019 data excluded from the 1-year analysis, as data for the full year were not available.

#### 2.3.5 Regression analysis of monthly incidence by species

Negative binomial regression models were fitted to the monthly probable rabies cases observed among all species, among domestic animals only and among wildlife only. Linear splines were used within the regression analyses where visual inspection of the data suggested a change in trend. The correlation between the monthly time series of cases in domestic dogs and in jackals was also examined, evaluating lags of 0 to 11 months for both time series.

#### 2.3.6 Logistic regression of cases in relation to population composition

We further examined whether the proportion of wildlife cases within a district was related to their relative availability within the susceptible population, focusing on only dogs and jackals (95% of all cases) and including data from other districts where cases had been traced using the same methods, specifically from Serengeti district (cases between January 2002 and June 2019), Ngorongoro district (January 2002 and March 2019) and Pemba Island (January 2010 and January 2019). The four districts of Pemba Island were considered a single population given the small numbers of dogs and limited geographical area.

The susceptible population (jackals and dogs) was estimated as follows. Jackals were assigned to grid cells at a density of 0.3 per km^2^ for all districts following a literature review (Durant et al., 2011; Maddox, 2003; Yarnell et al., 2013), except to cells with human population density below 2.5 per km^2^ or over 500 per km^2^ (using gridded population data from: https://www.worldpop.org/). Areas with lower human densities were excluded assuming negligible case detection from these largely uninhabited settings; the upper density limit was applied assuming unsuitable habitat. Alternative lower limits of 1.25 and 5 humans per km^2^ were explored as were alternative jackal densities of 0.15 and 0.50 jackals per km^2^. Dog numbers were estimated from post-vaccination transect data (Sambo et al., 2018). To account for dog vaccination on the availability of susceptible animals, three scenarios were applied to the estimated dog populations: (a) Zero vaccination coverage, (b) Median coverage recorded during the period and (c) Maximum recorded coverage. The combined susceptible (jackal and dog) population for each district was estimated. Using logistic regression, the proportion of probable cases (those in both dogs and jackals) that occurred in jackals was regressed against the proportion of the susceptible population consisting of jackals to assess evidence for a relationship.

All analyses were undertaken using the R statistical computing language (R Core Team, 2018).

## 3 Results

Over the 9-year study period, 688 human exposures to probable rabid animals were recorded within the Lindi and Mtwara regions. Of these exposures, 47 (6.8%) resulted in death due to probable rabies (none were laboratory confirmed). The number of probable animal rabies cases recorded over the same period was 549, comprising 313 cases (57.0%) in domestic animals and 236 (43.0%) in wildlife (Table 1). Only two of the animal rabies cases were laboratory confirmed. Domestic dogs accounted for the majority of human exposures to probable rabid animals (389/688, 56.5%) but jackals were responsible for a large proportion of the remaining exposures (262/688, 38.1%; Table 1). The highest incidence of exposures was found in Kilwa district (mean of 6.7/100,000 people/year), with a cluster of dog bites in 2018/19 (Figure 2). Mtwara Rural had the highest incidence of wildlife exposures (mean of 4.0/100,000 people/year). The incidence of human exposures by species and district are reported in the Supporting Information (Table S3).

**Figure 3:**
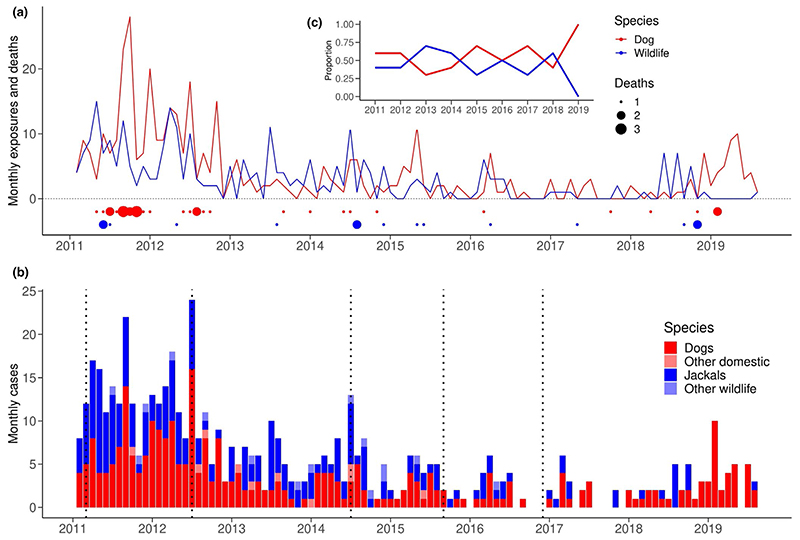
Probable animal rabies cases, human rabies exposures and deaths by species from January 2011 to July 2019. (a) Exposures (lines) and deaths (dots scaled by the number) from domestic dogs (red) and wildlife, mainly jackals (blue). (b) Cases in domestic dogs (red), jackals (blue), domestic cats (pink) and other wildlife (pale blue). Dashed lines indicate vaccination campaigns from 2011 to 2016. (c) The proportion of human exposures by species (dogs in red, wildlife in blue)

Over time, probable rabies exposures from all species, and probable animal rabies cases in both domestic animals and wildlife decreased across all districts (Figure 3). Most human rabies deaths and exposures occurred in 2011 (18 deaths, 218 exposures), while fewest exposures (15) were recorded in 2017 and fewest deaths in 2016 and 2019 (one each year). Probable animal rabies cases declined from 2011 to 2017, but then began to rise in 2018. In the first 2 years of the study, dogs accounted for over 1.5 times more human rabies exposures than wildlife. However, from 2013 onwards, the number of human exposures from domestic dogs and wildlife became more even, with wildlife accounting for more exposures than dogs in 2013 and 2014 (Figure 3). Throughout the study period, there were districts with wildlife cases detected in the absence of domestic dog cases and vice versa. Probable rabies cases were identified in mainly inhabited areas (Figure 2).

### 3.1 Parameter estimates

The best-fitting distribution to the serial intervals recorded from Serengeti District, northern Tanzania was a log-normal with mu and sigma parameters of 2.80 and 0.97, respectively, corresponding to a mean interval of 26.3 days with a standard deviation of 25.4 days (Figure S2a). Using a 50-m upper limit for the interval censoring of recorded zero values, the best-fitting distribution for the distance kernel was a gamma distribution with shape and scale parameters of 0.34 and 2,560, respectively, giving a mean distance of 873 m with a standard deviation of 1,495 m (Figure S2b). Likelihood ratio tests indicated no significant differences in how well the parameters derived from the Serengeti data fitted to the south-east Tanzania data compared to those derived from the south-east Tanzania data alone (*p* = 0.171 for the serial interval distributions, and *p* = 0.080 and *p* = 0.128 for the distance kernel with an upper limit of 50 and 100 m for the interval censoring respectively). Details of the other fitted distributions and those using a 100-m upper limit for interval censoring are in the Supporting Information (Figures S3–S5).

### 3.2 Transmission trees

Of the 549 inferred transmissions, 304 had values within the 99th percentile of the distributions for the serial interval and the convolved distance kernel and were included in subsequent analysis.

### 3.3 Within- and between-species transmission

Dog-to-dog transmission events were inferred to occur most frequently and represented 123 of 304 transmission events (40.5%, 95% confidence interval (CI) 35.2%–45.7%). Wildlife-to-wildlife transmission was the next most frequent accounting for 99 of 304 transmission events (32.6%, 95% CI 27.6%–37.8%). Dog-to-wildlife and wildlife-to-dog transmission events were inferred to occur with similar frequency at 10.5% (95% CI 7.2%–14.1%) and 13.5% (95% CI 10.2%–17.1%) of transmission events respectively (Table 2). Fisher’s exact test values were highly significant, with *p*-values of <0.001 for all of the 1,000 contingency tables of inferred transmission, suggesting that the observed patterns did not occur by chance mixing of species. Similar results were observed for all the scenarios examined as part of sensitivity analyses. Using lower cut-offs (95th percentile) for the serial interval and distance kernel to assign likely, direct transmission events resulted in a slight increase in the percentage of dog-to-dog transmissions and a slight decrease in the percentage of dog-to-wildlife and wildlife-to-dog transmissions. Very little effect was seen on the percentage of wildlife-to-wildlife transmission (Table S4). Subsampling dog cases appeared to reduce the percentage of transmission inferred to occur from dog-to-dog (and correspondingly increased wildlife transmission as a percentage of all transmission), but did not affect interspecific transmission (Appendix S4 and Figure S6).

### 3.4 Chains of transmission and cluster size

**Figure 4:**
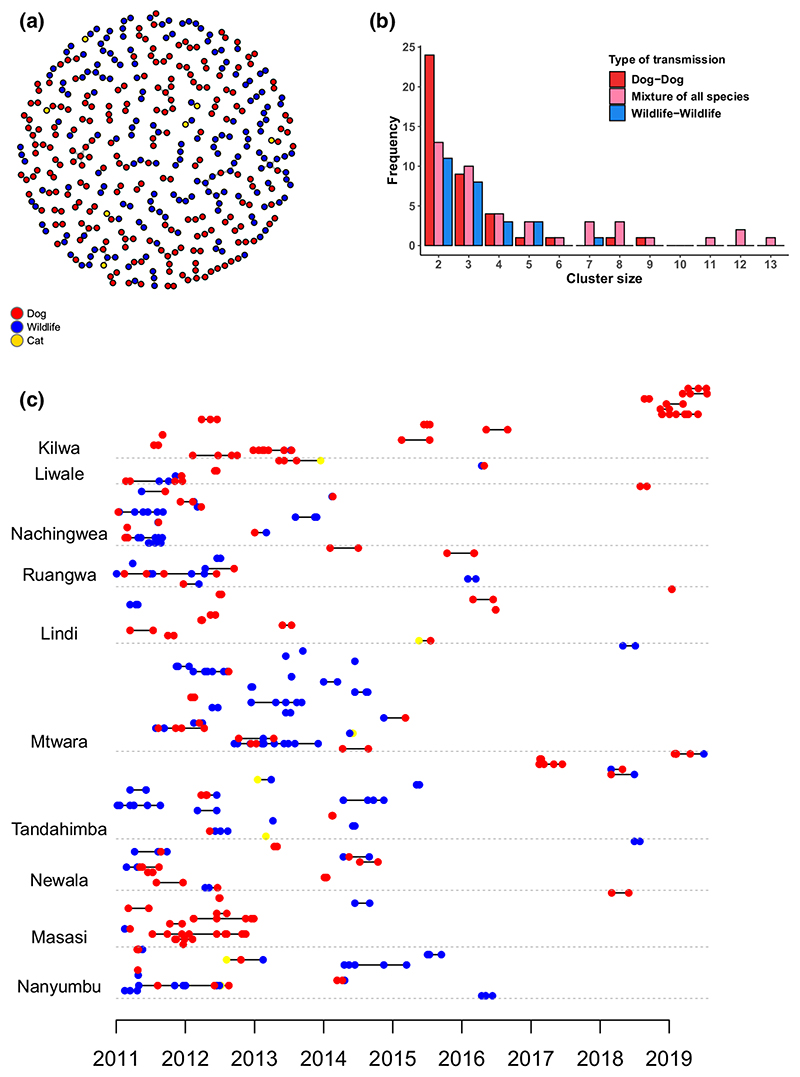
Inferred transmission chains and corresponding clusters according to species involved. Inferred transmission events within the 99th percentile of the serial interval and convolution of two distance kernel distributions (156 days and 9,803 m), using the single most likely progenitor for each case. (a) Inferred transmission chains showing domestic dogs (red), wildlife (blue) and cats (yellow); (b) Frequency and composition of clusters by size; and (c) inferred transmission chains by date of cases and district (coloured as for (a))

**Figure 5:**
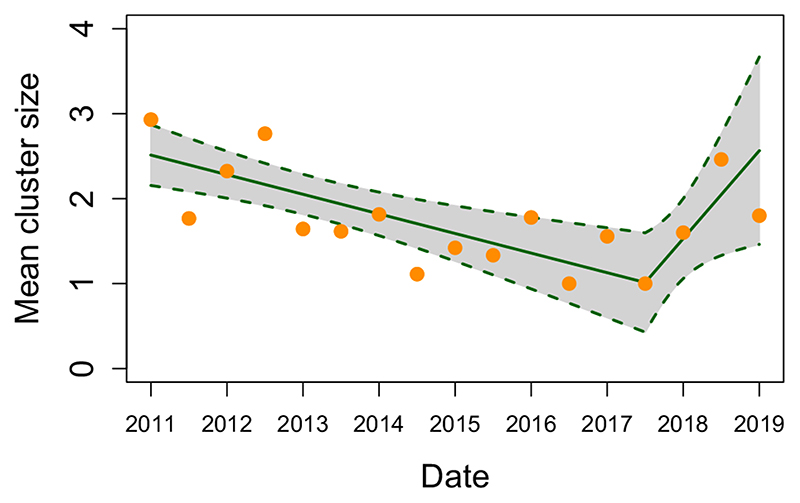
Trend in mean cluster size per 6-month period from January 2011 until July 2019. Mean cluster sizes for each 6-month period (orange) with the fitted linear spline regression (green) and associated 95% CI. A statistically significant downward trend in mean cluster size was observed over the initial 6-and-a-half years of the study (*p* = 0.001, reduction in mean cluster size of 0.12 (95% CI: 0.06–0.17) per 6-month period), followed by a statistically significant increase from July to December 2017 (*p* = 0.028, increase in mean cluster size of 0.52 (95% CI: 0.10–0.93) per 6-month period)

Chains of transmission were constructed from the most likely inferred progenitors and indicated clusters of dog-to-dog and wildlife-to-wildlife transmission (Figure 4). Clusters composed solely of dog-to-dog transmission were observed more frequently than those of solely wildlife-to-wildlife transmission. The largest clusters involved a mixture of species: the largest cluster of 13 comprised two dogs and 11 jackals, while clusters of 12 comprised 11 dogs and one jackal in one chain and two dogs and 10 jackals in the other. One hundred and sixty-three cases could not be linked to other cases (>99th percentile of the serial interval or convolved distance kernel distribution): 95 cases in dogs (58.3%), 65 in wildlife (40.5%) and three in cats. Chains of transmission occurred more frequently and were longer during the first half of the study (Figure 4c). While almost all districts had wildlife cases, some appeared to have very little sustained wildlife transmission (Kilwa, Liwale, Lindi, Masasi), whereas others had much greater wildlife involvement (Mtwara, Tandahimba). A weighted linear spline regression with a single knot at the July–December 2017 6-month period demonstrated a statistically significant decrease in mean cluster size over the first 6-and-a-half years of the study (*p* = 0.001, decrease in mean cluster size of 0.12 per 6-month period, 95% CI: 0.06–0.17), followed by a statistically significant increase (*p* = 0.028, increase in mean cluster size of 0.52 per 6-month period, 95% CI: 0.10–0.93, Figure 5). Sensitivity analyses using 3-month and 1-year periods were consistent, with statistically significant decreases in cluster size over the first 6-and-a-half and 7 years of the study respectively. After the initial period, cluster size increases significantly using 3-month periods, but this increase is not significant using 1-year periods likely due to the omission of the incomplete 2019 data.

Of the 32 cases where the progenitor was known, the correct biting animal was not always assigned with the highest bootstrap probability. In 16 of 32 cases, the biting animal was correctly identified with less than 5% bootstrap probability. All of these cases involved dogs that were part of clusters within households. The algorithm identified a different dog but always one within the same household and cluster, meaning the assigned species-to-species transmission was correct.

### 3.5 Regression analysis of monthly incidence

Negative binomial regression models with a linear spline at August 2017 supported a statistically significant downward trend in monthly probable rabies cases between January 2011 and August 2017 (*p* < 0.001, 3.1% reduction per month in all species [95% CI: 2.6%–3.6%]) and in domestic animals only (*p* < 0.001, 3.1% reduction per month [95% CI: 2.4%–3.7%]) when fitted to cases from all species or from domestic animals only. The change in slopes from August 2017 was statistically significant in both models (*p* < 0.001, 5.5% increase per month in all species [95% CI: 2.9%–8.1%]; 8.2% increase in domestic animals only [95% CI: 5.1%–11.3%]). For probable cases in wildlife, the slope did not change significantly (*p* = 0.63), therefore a single trend was maintained (3.0% reduction per month in wildlife [95% CI: 2.4%–3.6%]). Plots of the fitted models are shown in the Supporting Information (Figure S7).

When assessing correlations between monthly domestic dog and jackal cases with lags ranging from 0 to 11 months, all scenarios had significant positive correlation coefficients. The largest coefficients occurred with no lag applied between monthly cases and when jackal cases were leading with a 4-month lag applied to dog cases (coefficient 0.525, *p* < 0.001 for both lags). Full results are presented in Table S5.

### 3.6 Logistic regression of cases in relation to population composition

**Figure 6:**
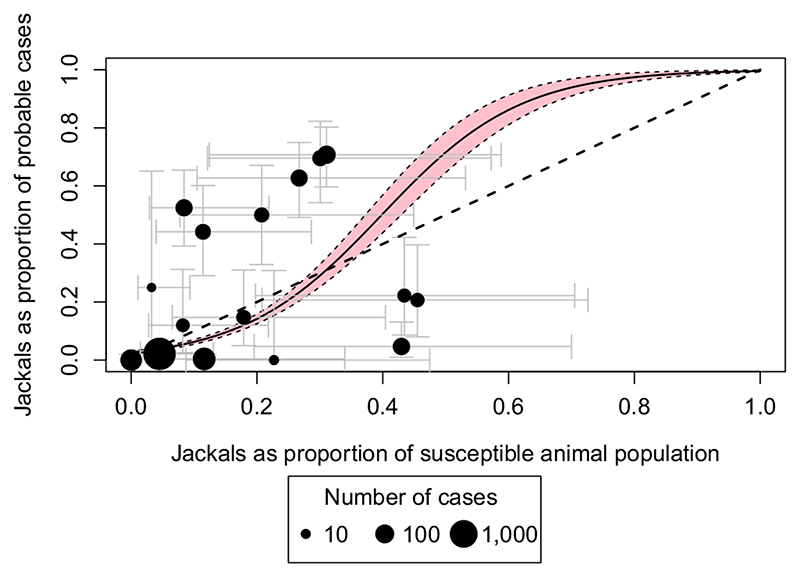
Relationship between the proportion of jackals in the susceptible animal population and the proportion of probable rabies cases in jackals. The susceptible population consists of jackals and unvaccinated dogs assuming the median level of vaccination coverage. Probable rabies cases refer to those in jackals and domestic dogs only. Dots represent the 16 districts in this analysis, scaled by the log10 cases in the district. Grey bars represent 95% confidence intervals (CIs). The CIs around the proportion of probable cases in jackals are exact binomial 95% CIs and were calculated keeping jackal estimates and levels of vaccination coverage constant but incorporating the lower and upper limits of the 95% CIs of the dog number estimates. The fitted logistic regression line is shown in black with the associated 95% CI in pink

Logistic regression suggested a statistically significant positive relationship (*p* < 0.001, Figure 6) between jackals as a proportion of the susceptible population and the proportion of probable cases that were in jackals (when jackals were distributed across areas with >2.5 and <500 humans/km^2^). Results obtained using minimum and maximum district-level vaccination coverages, different cut-offs for human densities used for estimating jackal populations and different jackal density estimates were all statistically significant (Appendix S5 and Figure S8).

## 4 Discussion

Less than a decade remains to achieve the ‘Zero by Thirty’ global target of zero deaths from dog-mediated rabies by 2030. Here we present data from a previously unstudied area of south-east Tanzania following the introduction of large-scale dog vaccination. We examine whether wildlife could present an obstacle to rabies elimination and ‘Zero by Thirty’, under the hypothesis that if domestic dogs are the sole maintenance host, then control strategies directed at dogs alone should interrupt transmission. Throughout the 9-year study, most human rabies exposures and probable animal rabies cases were detected in domestic dogs. However, wildlife were a key source of human rabies exposures and comprised a large proportion of probable animal rabies cases. Wildlife-to-wildlife transmission accounted for approximately one third of inferred transmissions, and cross-species transmission among dogs and jackals was inferred to occur frequently. Both probable animal rabies cases and human rabies exposures decreased during the period of dog vaccinations, as did the size of inferred transmission clusters among all species. We attribute the initial decreased transmission observed across all species to the implementation of widespread dog vaccination and suggest that the increased cases in domestic dogs in 2018/19 resulted from waning herd immunity, coincident with cessation of widespread dog vaccination. While domestic dogs are the main reservoir host for the maintenance of rabies in south-east Tanzania, our data suggest that wildlife can sustain transmission chains and pose a substantive public health risk. In contrast to work from northern Tanzania showing that domestic dogs are the only species in which rabies appears capable of persisting (Lembo et al., 2008), here we find much greater involvement of jackals, but still conclude that targeting dogs through mass vaccination should eliminate rabies in this area.

One challenge faced during this study was limited information on jackal populations. We extrapolated jackal numbers using density estimates from studies elsewhere in Africa, but this approach does not incorporate geographical population differences. More accurate jackal numbers would underpin a more confident assessment of the relationship between the susceptible population and cases. This, in turn, could provide further insight into which species drive transmission and whether assortative mixing underpins transmission pathways or if transmission depends more on the availability of susceptible animals regardless of species. Additional data will be needed to conclude whether jackals can maintain RABV independently over the longer term. Our conclusion that where more jackals are present, rabies incidence in these populations is correspondingly higher is robust to the range of estimates of jackal density considered.

A further limitation related to case detection and confirmation. A low proportion of probable rabies cases were confirmed through laboratory diagnosis. Of the 549 clinically diagnosed animals, samples were collected in only two cases, both of which tested positive. The low rate of sample submission was primarily due to delays in reporting across this large area such that on follow-up the animal had been lost or the carcass decomposed. Low rates of sample submission also meant that genomic data were not available. We considered that assigned progenitors in 304 of the 549 probable cases were likely to represent direct transmission. This suggests that despite intensive effort, over one third of cases were not observed (i.e. no progenitor found for the remaining 245 cases). We also note that case locations were associated with uncertainty (31.5% within an estimated 0–2 km radius of the precise location, 22.0% within 5 km and 5.1% within 5–10 km). Despite unobserved transmission, our results were nonetheless robust under sensitivity analyses.

Genetic sequencing should prove useful in resolving transmission chains by determining whether RABV lineages include cases in both dogs and wildlife and for identifying introductions of RABV via human-mediated dog movement. Although several RABV lineages have been detected across Tanzania, there is currently no evidence of species-specific lineage associations (Brunker et al., 2015; Lembo et al., 2008). Translocations of dogs has been shown to be important in the spread of RABV (Denduangboripant et al., 2005) and genomic approaches have revealed substantial human-mediated RABV movement in Tanzania (Brunker et al., 2015), which may explain how some apparently unconnected clusters and cases arose.

Overall, our study provides insights into the epidemiology of rabies in multi-host communities and highlights the potential importance of wildlife as sources of rabies exposure. Our data highlight the frequent transmission of rabies from sparsely distributed domestic dog populations, to and from sympatric wildlife, specifically jackals (Figure 2). Yet even in areas with relatively high proportions of wildlife cases, domestic dog vaccination still reduced the risk to humans. Maintaining dog vaccination campaigns in LMICs is challenging, and we show that if vaccination campaigns are not maintained, resurgence of rabies can rapidly occur. Herd immunity wanes quickly with high demographic turnover in the dog population, and infection circulating in nearby populations can seed introductions (Hampson et al., 2009; Zinsstag et al., 2017). Continued dog vaccination is needed to eliminate rabies from south-east Tanzania and should shed more light on the involvement of wildlife in rabies maintenance. A useful future extension would be to estimate the type reproduction number for dogs and wildlife in these regions. The type reproduction number can be used to estimate the control effort required to eliminate an infectious disease within a maintenance community when control is targeted at a subset of hosts (Roberts & Heesterbeek, 2003). Its application to rabies control was recently illustrated in relation to vaccination strategies targeting owned, free-roaming and stray dogs (Leung & Davis, 2017). While spillovers from RABV maintenance hosts into other species are common, most do not result in ongoing transmission (Mollentze et al., 2020). However, host shifts (establishment of novel cycles of transmission in new host species) occasionally occur and have important implications for control. Although the mechanisms that drive host shifts are poorly understood, if RABV continues to circulate within domestic dogs in south-east Tanzania, spillover to wildlife is likely and opportunities for a host shift remain. The establishment of sustained transmission within wildlife would have a serious impact on the effectiveness of control strategies currently focused on dog vaccination, which lends further urgency to eliminating rabies in dogs now.

## 5 Conclusions

Even in this area with relatively high proportions of wildlife rabies cases and evidence of cross-species transmission, our work indicates that domestic dog vaccination appears to be effective in reducing exposure risks in humans and decreasing rabies incidence among all species. The importance of sustained annual vaccinations is highlighted by the observed increase in probable dog cases following the cessation of widespread vaccination campaigns in 2017. This increase in domestic dog rabies and likely subsequent increase in wildlife rabies represents a significant public health threat. These findings have implications for Tanzania’s National Rabies Control strategy and suggest that focusing on domestic dog vaccination will have major public health benefits, and if sustained and coordinated may eliminate RABV. Ongoing effective surveillance will be essential to monitor the impacts of dog vaccination, which needs scaling up to reach the ‘Zero by Thirty’ target. Engaging the wildlife sector and building genomic surveillance capacity in particular would further resolve transmission dynamics within domestic dogs and wildlife and inform progression towards elimination.

## Supplementary Material

Fig S1

Fig S2

Fig S3

Fig S5

Fig S6

Fig S7

Fig S8

Supplementary text

## Figures and Tables

**Table 1:** Probable animal rabies cases, human exposures and human rabies deaths by infecting species detected from January 2011 to July 2019 in Lindi and Mtwara regions. In addition, four people died from bite injuries from probable rabid hyenas (3) and a probable rabid jackal (1)

Group	Species	Probable animal rabies cases(%)	Human rabies exposures by species (%)	Human rabies deaths by infecting species (%)
Domestic animals	Dog	303 (55.2)	389 (56.5)	32 (68.1)
	Cat	10 (1.8)	12 (1.7)	0 (0)
Wildlife	Jackal	221 (40.3)	262 (38.1)	12 (25.5)
	Hyena	8 (1.5)	16 (2.3)	3 (6.4)
	Honey badger (*Mellivora capensis*)	5 (0.9)	6 (0.9)	0 (0)
	Leopard (*Panthera pardus*)	2 (0.4)	3 (0.4)	0 (0)

**Table 2:** Number and percentage of inferred direct transmissions between species. Results from the analysis of inferred transmissions with values within the 99th percentile of the serial interval and convolution of two distance kernel distributions (156 days and 9,803 m respectively)

	Transmissions (% of total)	
Transmission	Median	Bootstrap 95% confidence interval
Dog–dog	123 (40.5)	107–139 (35.2–45.7)
Dog–wildlife	32 (10.5)	22–43 (7.2–14.1)
Wildlife–dog	41 (13.5)	31–52 (10.2–17.1)
Wildlife–wildlife	99 (32.6)	84–115 (27.6–37.8)
Cat–dog	2 (0.7)	0–6 (0.0–2.0)
Dog–cat	1 (0.3)	0–4 (0.0–1.3)
Cat–wildlife	1 (0.3)	0–3 (0.0–1.0)
Wildlife–cat	4 (1.3)	0–8 (0.0–2.6)
Cat–cat	0 (0.0)	0 (0.0–0.0)

## Data Availability

The code for our analyses are available on our public Github public repository (https://github.com/LushasiK/Stz_rabies_reservoir), as well as anonymized data which allow replication of most results. The anonymized data are available via the Dryad Digital Repository https://doi.org/10.5061/dryad.bg79cnpbg (Lushasi et al., 2021). Our full dataset contains georeferences that are available on request from the authors following ethical approval for secondary reuse.

## References

[R1] Bellan SE, Cizauskas CA, Miyen J, Ebersohn K, Küsters M, Prager KC, Van Vuuren M, Sabeta C, Getz WM (2012). Black-backed jackal exposure to rabies virus, canine distemper virus, and Bacillus anthracis in Etosha National Park, Namibia. Journal of Wildlife Diseases.

[R2] Bingham J, Foggin CM, Wandeler AI, Hill FWG (1999). The epidemiology of rabies in Zimbabwe. 2. Rabies in jackals *Canis adustus* and *Canis mesomelas*. Onderstepoort Journal of Veterinary Research.

[R3] Brunker K, Marston DA, Horton DL, Cleaveland S, Fooks AR, Kazwala R, Ngeleja C, Lembo T, Sambo M, Mtema ZJ, Sikana L (2015). Elucidating the phylodynamics of endemic rabies virus in eastern Africa using whole-genome sequencing. Virus Evolution.

[R4] Courtin F, Carpenter TE, Paskin RD, Chomel BB (2000). Temporal patterns of domestic and wildlife rabies in central Namibia stock-ranching area, 1986-1996. Preventive Veterinary Medicine.

[R5] Denduangboripant J, Wacharapluesadee S, Lumlertdacha B, Ruankaew N, Hoonsuwan W, Puanghat A, Hemachudha T (2005). Transmission dynamics of rabies virus in Thailand: Implications for disease control. BMC Infectious Diseases.

[R6] Durant SM, Craft ME, Hilborn R, Bashir S, Hando J, Thomas L (2011). Long-term trends in carnivore abundance using distance sampling in Serengeti National Park, Tanzania. Journal of Applied Ecology.

[R7] Hampson K, Coudeville L, Lembo T, Sambo M, Kieffer A, Attlan M, Barrat J, Blanton JD, Briggs DJ, Cleaveland S, Costa P (2015). Estimating the global burden of endemic canine rabies. PLOS Neglected Tropical Diseases.

[R8] Hampson K, Dushoff J, Cleaveland S, Haydon DT, Kaare M, Packer C, Dobson A (2009). Transmission dynamics and prospects for the elimination of canine Rabies. PLoS Biology.

[R9] Haydon DT, Cleaveland S, Taylor LH, Laurenson MK (2002). Identifying reservoirs of infection: A conceptual and practical challenge. Emerging Infectious Diseases.

[R10] Hikufe EH, Freuling CM, Athingo R, Shilongo A, Ndevaetela E-E, Helao M, Shiindi M, Hassel R, Bishi A, Khaiseb S, Kabajani J (2019). Ecology and epidemiology of rabies in humans, domestic animals and wildlife in Namibia, 2011-2017. PLOS Neglected Tropical Diseases.

[R11] Lembo T, Hampson K, Haydon DT, Craft M, Dobson A, Dushoff J, Ernest E, Hoare R, Kaare M, Mlengeya T, Mentzel C (2008). Exploring reservoir dynamics: A case study of rabies in the Serengeti ecosystem. Journal of Applied Ecology.

[R12] Leung T, Davis SA (2017). Rabies vaccination targets for stray dog populations. Frontiers in Veterinary Science.

[R13] Lushasi K, Hayes S, Ferguson EA, Changalucha J, Cleaveland S, Govella NJ, Haydon DT, Maganga S, Mchau GJ, Mpolya EA, Mtema Z (2021). Data from: Reservoir dynamics of rabies in southeast Tanzania and the roles of cross-species transmission and domestic dog vaccination. Dryad Digital Repository.

[R14] Maddox TM (2003). The ecology of cheetahs and other large carnivores in a pastoralist-dominated buffer zone. Dissertation.

[R15] Moagabo KT, Monyame KB, Baipoledi EK, Letshwenyo M, Mapitse N, Hyera JMK (2009). A retrospective longitudinal study of animal and human rabies in Botswana 1989-2006. Onderstepoort Journal of Veterinary Research.

[R16] Mollentze N, Streicker DG, Murcia PR, Hampson K, Biek R (2020). Virulence mismatches in index hosts shape the outcomes of cross-species transmission. Proceedings of the National Academy of Sciences of the United States of America.

[R17] Molyneux D, Sankara DP (2017). Guinea worm eradication: Progress and challenges—should we beware of the dog?. PLoS Neglected Tropical Diseases.

[R18] Mpolya EA, Lembo T, Lushasi K, Mancy R, Mbunda EM, Makungu S, Maziku M, Sikana L, Jaswant G, Townsend S, Meslin F-X (2017). Toward elimination of dog-mediated human rabies: Experiences from implementing a large-scale demonstration project in southern Tanzania. Frontiers in Veterinary Science.

[R19] Mtema Z, Changalucha J, Cleaveland S, Elias M, Ferguson M, Halliday JEB, Haydon DT, Jaswant G, Kazwala R, Killeen GF, Lembo T (2016). Mobile phones as surveillance tools: Implementing and evaluating a large-scale intersectoral surveillance system for rabies in Tanzania. PLoS Medicine.

[R20] Nadin-Davis SA, Velez J, Malaga C, Wandeler AI (2008). A molecular epidemiological study of rabies in Puerto Rico. Virus Research.

[R21] Pfukenyi DM, Pawandiwa D, Makaya PV, Ushewokunze-Obatolu U (2009). A retrospective study of wildlife rabies in Zimbabwe, between 1992 and 2003. Tropical Animal Health and Production.

[R22] R Core Team (2018). R: A language and environment for statistical computing.

[R23] Randall DA, Williams SD, Kuzmin IV, Rupprecht CE, Tallents LA, Tefera Z, Argaw K, Shiferaw F, Knobel DL, Sillero-Zubiri C, Laurenson MK (2004). Rabies in endangered Ethiopian wolves. Emerging Infectious Diseases.

[R24] Roberts MG, Heesterbeek JAP (2003). A new method for estimating the effort required to control an infectious disease. Proceedings of the Royal Society of London Series B: Biological Sciences.

[R25] Rupprecht CE, Hanlon CA, Hemachudha T (2002). Rabies re-examined. Lancet Infectious Diseases.

[R26] Sabeta CT, Mansfield KL, McElhinney LM, Fooks AR, Nel LH (2007). Molecular epidemiology of rabies in bat-eared foxes *Otocyon megalotis* in South Africa. Virus Research.

[R27] Sambo M, Hampson K, Changalucha J, Cleaveland S, Lembo T, Lushasi K, Mbunda E, Mtema Z, Sikana L, Johnson P (2018). Estimating the size of dog populations in Tanzania to inform rabies control. Veterinary Sciences.

[R28] Thomson GR, Meredith CD (1993). Rabies in bat-eared foxes in South Africa. The Onderstepoort Journal of Veterinary Research.

[R29] United Nation Development Programme (2018). Government of the United Republic of Tanzania, Ministry of Finance and Planning. Tanzania Human Development Report 2017.

[R30] Velasco-Villa A, Escobar LE, Sanchez A, Shi M, Streicker DG, Gallardo-Romero NF, Vargas-Pino F, Gutierrez-Cedillo V, Damon I, Emerson G (2017). Successful strategies implemented towards the elimination of canine rabies in the Western Hemisphere. Antiviral Research.

[R31] WHO (2018). WHO expert consultation on rabies. Technical report series.

[R32] WHO, FAO, & OIE (2015). Rationale for investing in the global elimination of dog-mediated human rabies.

[R33] Yarnell RW, Phipps WL, Burgess LP, Ellis JA, Harrison SWR, Dell S, MacTavish D, MacTavish LM, Scott DM (2013). The influence of large predators on the feeding ecology of two African mesocarnivores: The black-backed jackal and the brown hyaena. South African Journal of Wildlife Research.

[R34] Youde J (2013). Cattle scourge no more: The eradication of rinderpest and its lessons for global health campaigns. Politics and the Life Sciences.

[R35] Zinsstag J, Lechenne M, Laager M, Mindekem R, Naïssengar S, Oussiguéré A, Bidjeh K, Rives G, Tessier J, Madjaninan S, Ouagal M (2017). Vaccination of dogs in an African city interrupts rabies transmission and reduces human exposure. Science Translational Medicine.

[R36] Zulu GC, Sabeta CT, Nel LH (2009). Molecular epidemiology of rabies: Focus on domestic dogs *Canis familiaris* and black-backed jackals *Canis mesomelas* from northern South Africa. Virus Research.

