# Supplementary figures and images for: Reservoir dynamics of rabies in south-east Tanzania and the roles of cross-species transmission and domestic dog vaccination
Source: J Appl Ecol. Author manuscript; Available in PMC 2022 Feb 25. (PMC7612421; doi:10.1111/1365-2664.13983)

(A) Round 1

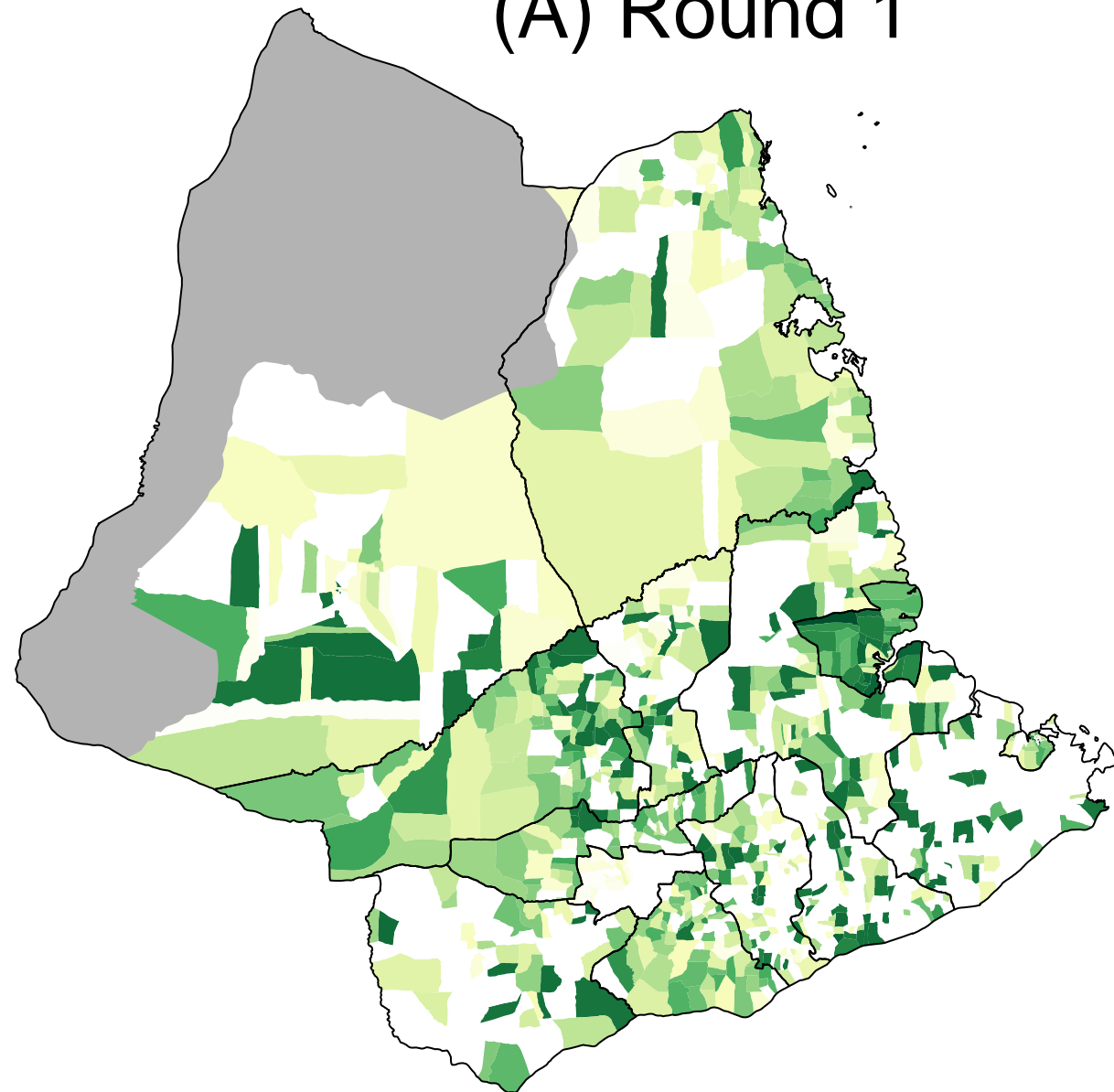

(B) Round 2

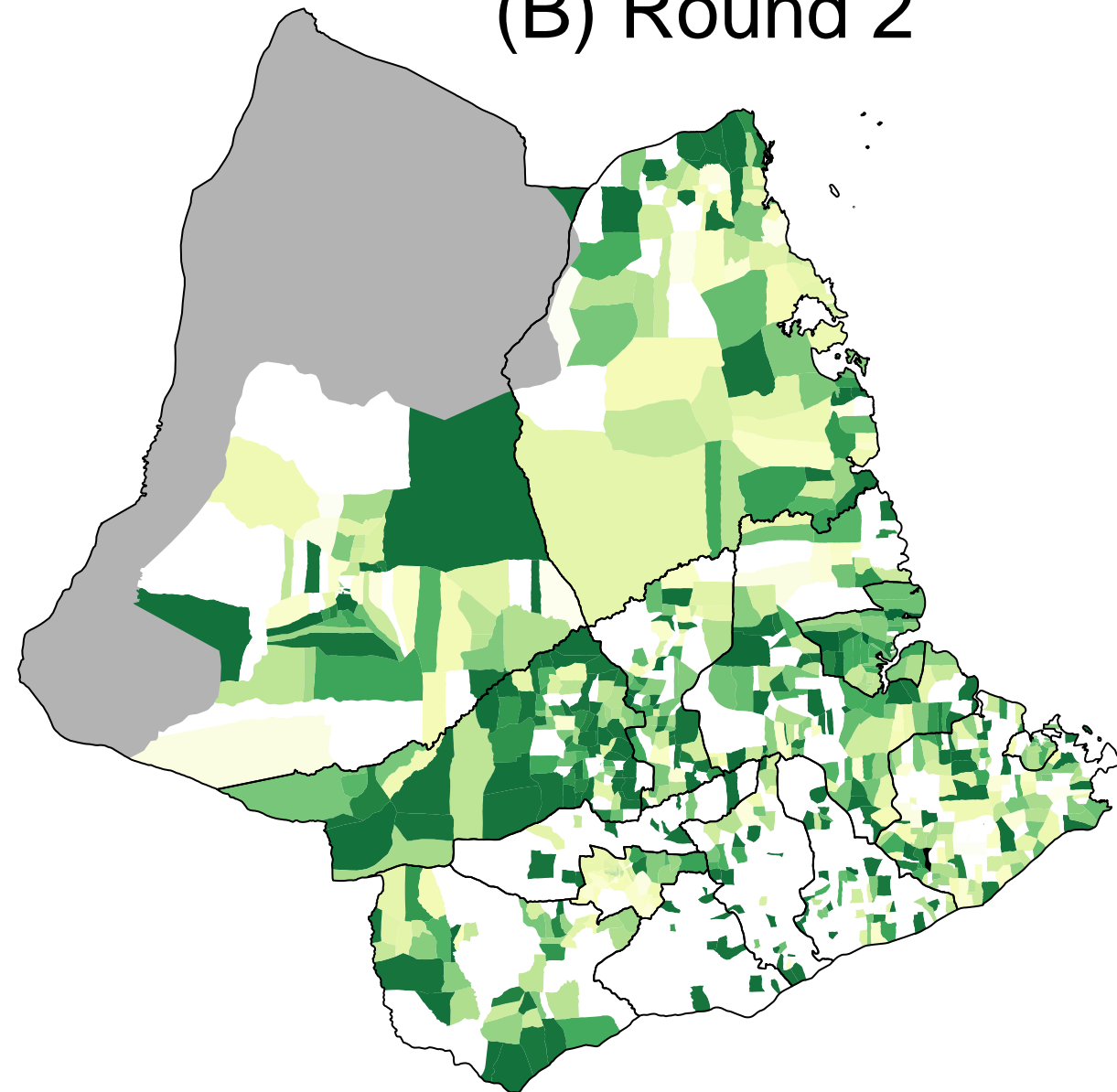

(C) Round 3

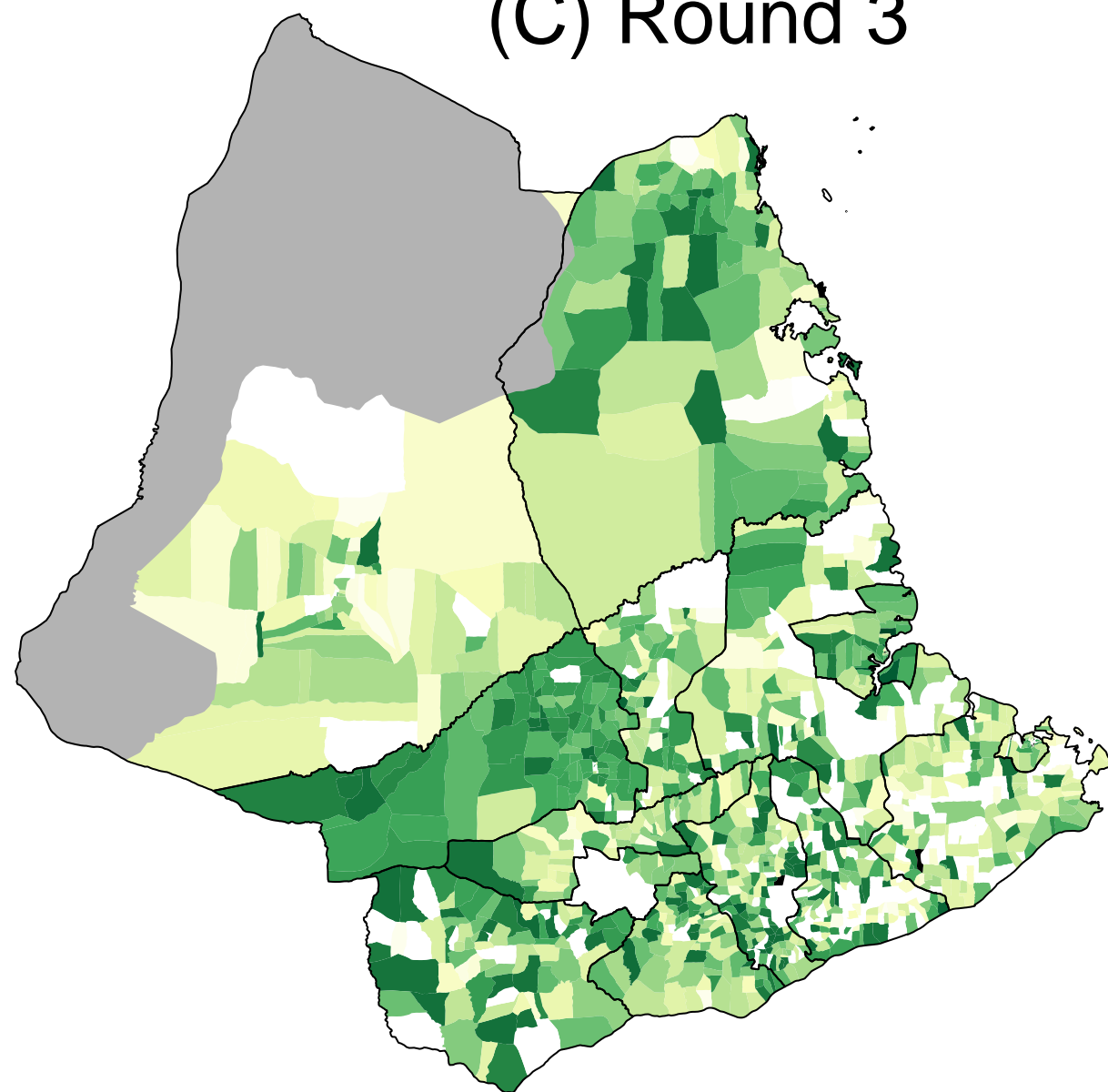

(D) Round 4

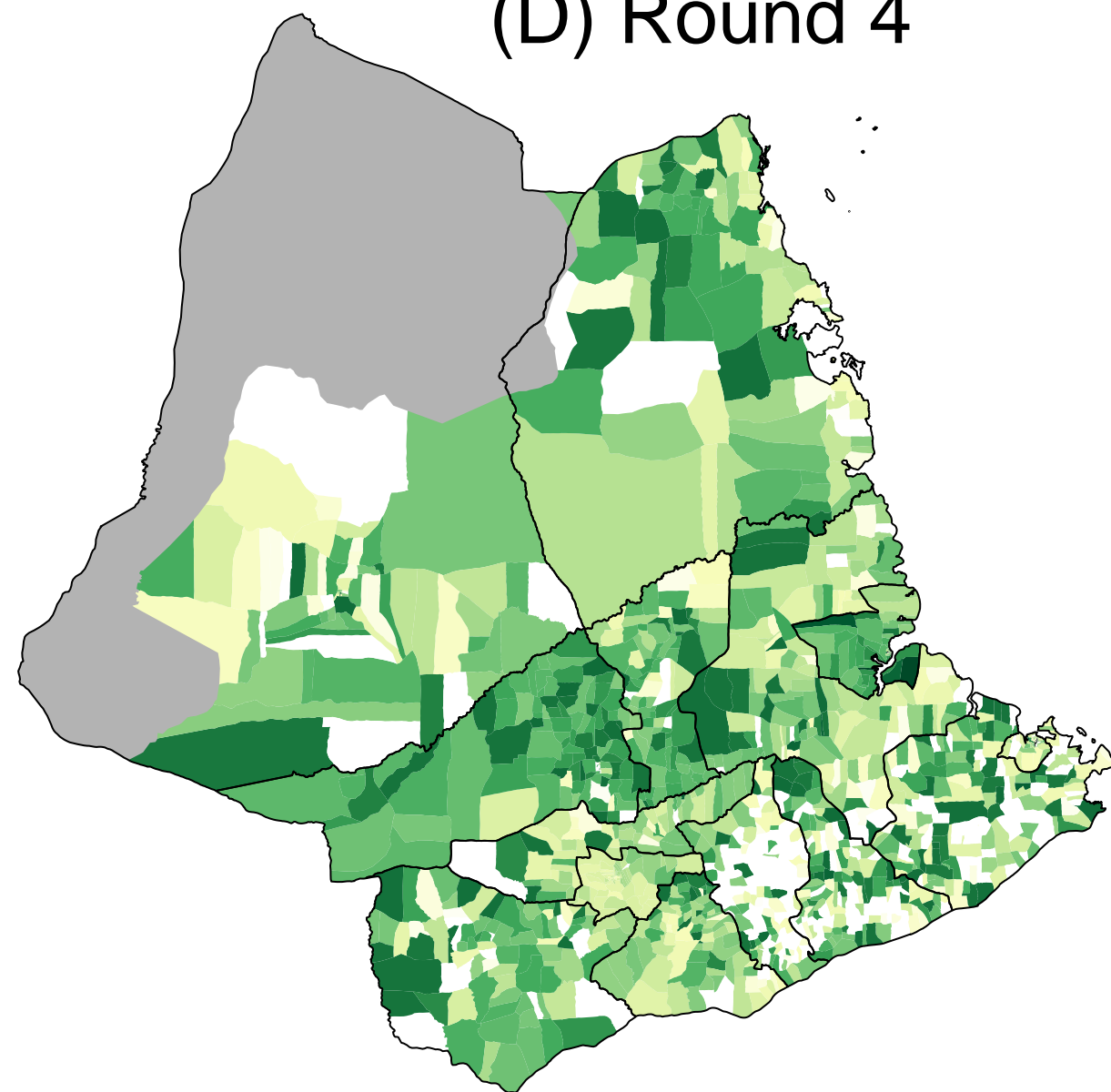

(E) Round 5

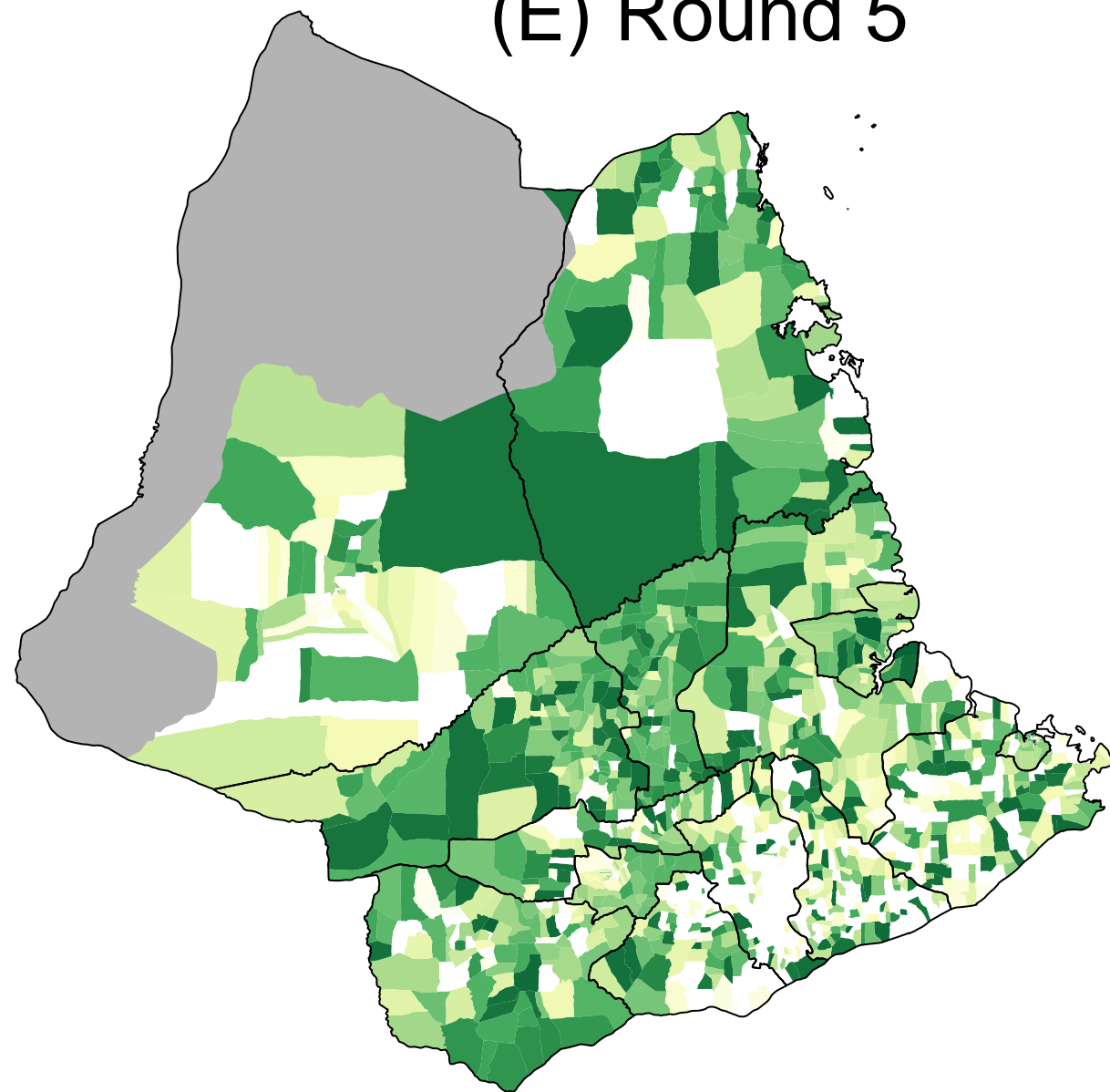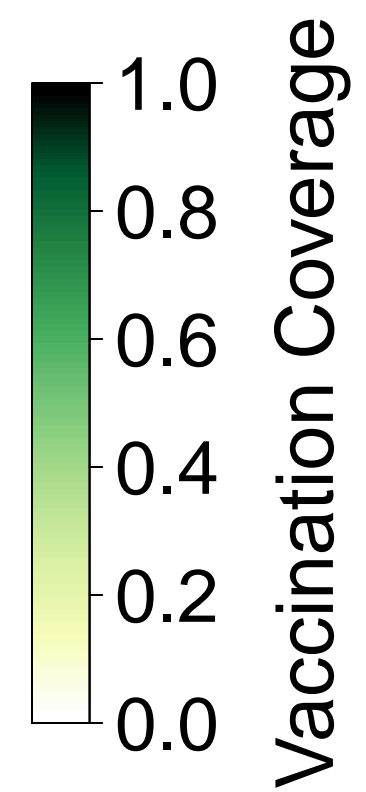

Supplement: Fig S1 [file EMS142064-supplement-Fig_S1.pdf]

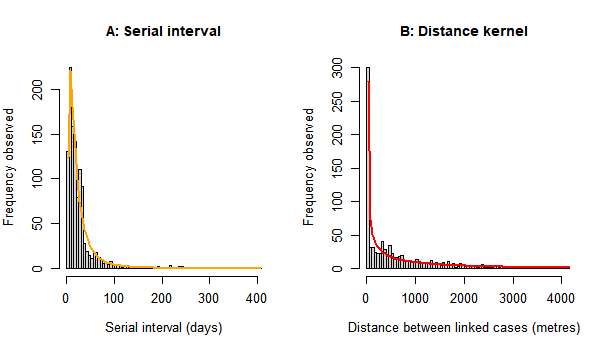

Supplement: Fig S2 [file EMS142064-supplement-Fig_S2.png]

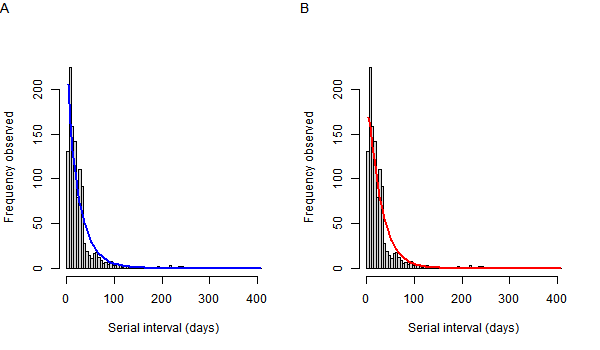

Supplement: Fig S3 [file EMS142064-supplement-Fig_S3.png]

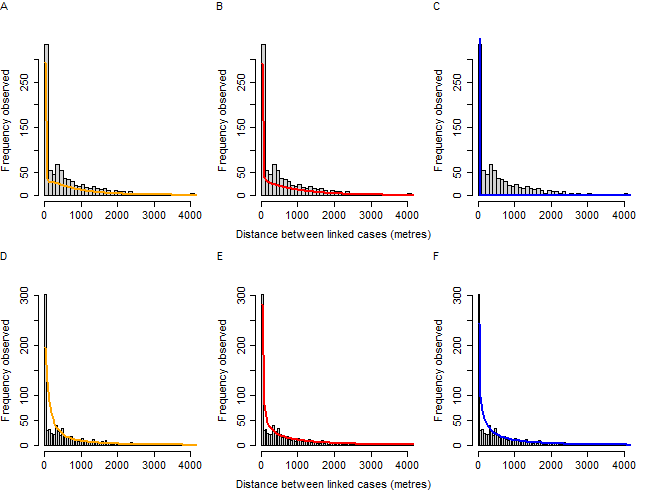

Supplement: Fig S5 [file EMS142064-supplement-Fig_S5.png]

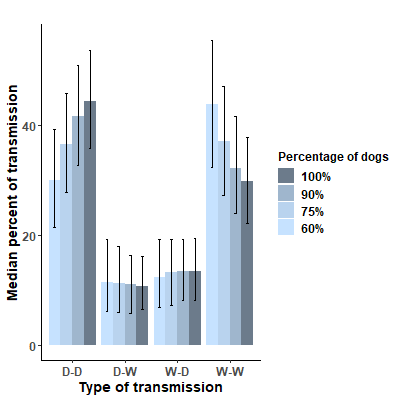

Supplement: Fig S6 [file EMS142064-supplement-Fig_S6.png]

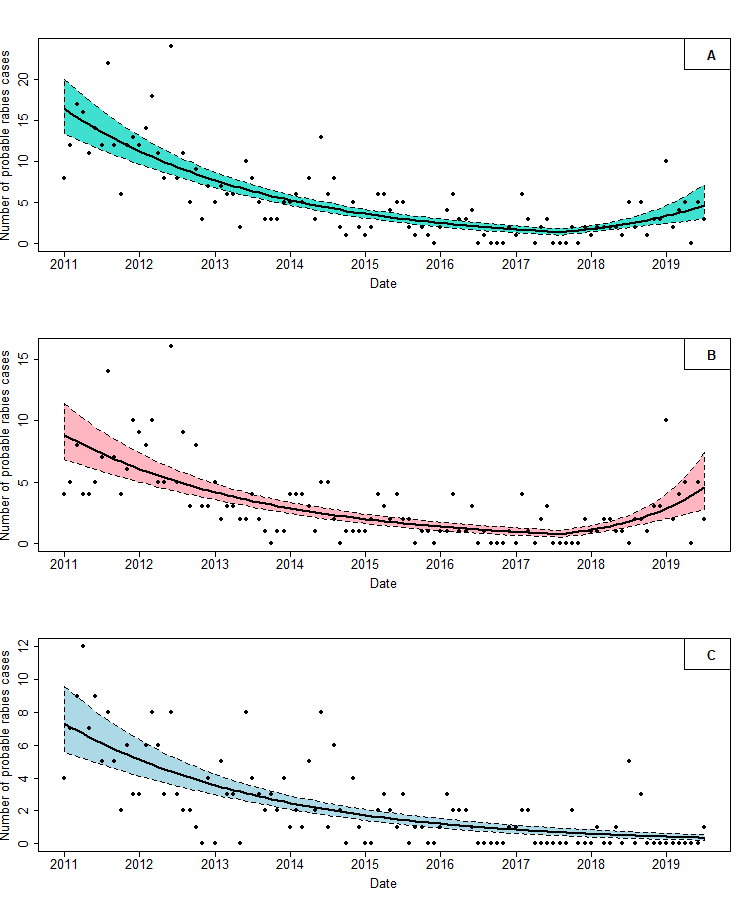

Supplement: Fig S7 [file EMS142064-supplement-Fig_S7.png]

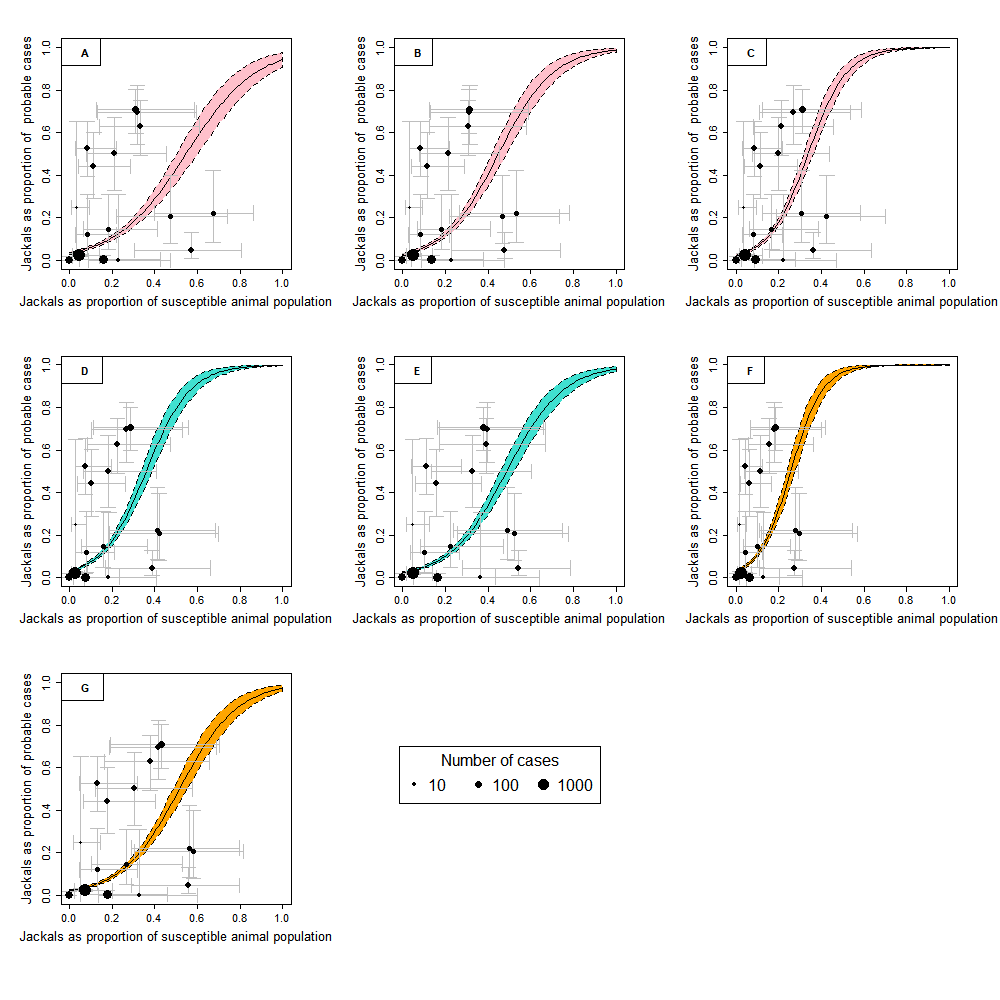

Supplement: Fig S8 [file EMS142064-supplement-Fig_S8.png]
