# Supplementary material for: Reservoir dynamics of rabies in south-east Tanzania and the roles of cross-species transmission and domestic dog vaccination
Source: J Appl Ecol. Author manuscript; Available in PMC 2022 Feb 25. (PMC7612421; doi:10.1111/1365-2664.13983)
Supplement: Supplementary text [file EMS142064-supplement-Supplementary_text.docx]

**Supplementary Information**

**Appendices**

S1: Description of mass dog vaccination campaigns

S2: Parameter estimation

S3: Sensitivity analyses

S4: Subsampling of species

S5: Logistic regression of cases in relation to population composition

**Supplementary Tables**

S1: Characteristics of the study districts and probable rabies cases identified

S2: Dog vaccination coverage by district

S3: Incidence of human rabies exposures

S4: Inferred transmissions between species

S5: Lagged correlation analysis of monthly cases

**Supplementary Figures**

S1: Vaccination coverage by vaccination round.

S2: best-fitting distributions for rabies serial intervals and distance kernels

S3: Alternative distributions for the serial interval

S4: Distance kernel with fitted distributions using interval censored data at 50m

S5: Distance kernel with fitted distributions using interval censored data at 100m

S6: Subsampled transmission tree analysis

S7: Regression analyses of probable animal rabies cases per month

S8: Sensitivity analyses examining relationship between cases in jackals and population composition

**Table S1: Characteristics of the study districts and probable rabies cases identified.** Ngorongoro, Serengeti districts and Pemba Island are outside of the study region but included for comparison, with cases reported between January 2002 - March 2019 for Ngorongoro; January 2010 - 2019 for Pemba and January 2002 - June 2019 for Serengeti.

| **District** | **Area in km^2^** | **Estimated human populated area in km^2^** | **Human population in 2018** | **Dog population in 2018 (95% CIs)** | **Domestic animal rabies from 2011-2019** | **Wildlife rabies from 2011-2019** |
| --- | --- | --- | --- | --- | --- | --- |
| **Kilwa** | 15,044 | 13,720 | 201,279 | 3,661 (1,181-11,346) | 61 | 3 |
| **Lindi Rural** | 5,984 | 5,900 | 204,865 | 2,261 (712-7182) | 23 | 6 |
| **Lindi Urban** | 1,052 | 1,032 | 83,195 | 1,345 (438-4,136) | 11 | 0 |
| **Liwale** | 34,296 | 14,008 | 96,427 | 2,236 (717-6,967) | 22 | 8 |
| **Masasi** | 3,992 | 3,976 | 266,393 | 6,167 (1,980-19,205) | 30 | 7 |
| **Masasi Township Authority** | 776 | 776 | 110,316 | 2,589 (831-8,065) | 23 | 3 |
| **Mtwara Rural** | 3,704 | 3,604 | 244,920 | 2,703 (853-8,570) | 24 | 60 |
| **Mtwara Urban** | 168 | 168 | 116,334 | 1,351 (441-4,143) | 7 | 2 |
| **Nachingwea** | 6,000 | 5,992 | 188,321 | 4,526 (1,454-14,093) | 22 | 38 |
| **Nanyumbu** | 5,176 | 5,164 | 162,050 | 3,883 (1,247-12,089) | 17 | 34 |
| **Newala** | 1,944 | 1,944 | 220,738 | 5,080 (1,631-15,823) | 24 | 19 |
| **Ruangwa** | 2,492 | 2,492 | 138,320 | 3,288 (1,056- 10,237) | 18 | 18 |
| **Tandahimba** | 2,040 | 2,036 | 244,394 | 7,629 (2,496-23,322) | 31 | 38 |
| **Ngorongoro** | 15,528 | 12,540 | 204,487 | 32,905 (8,406-128,805) | 368 | 37 |
| **Pemba** | 1,000 | 888 | 437,146 | 8213 (2,658-25,384) | 199 | 0 |
| **Serengeti** | 11,200 | 2,700 | 289,251 | 25,169 (7,905-80,138) | 3,521 | 149 |

**Appendix S1: Description of mass dog vaccination campaigns**

**Mass dog vaccination**: five dog vaccination campaigns were conducted in each district

between 2011 and 2017. Temporary vaccination stations were set up within villages at points chosen to be accessible to most villagers, often within a ward or village office, school or other central village location. Each vaccination point was operated by two livestock field officers (LFOs) and either a health worker or local primary school teacher. On arrival at the vaccination station, dogs were registered and their age, sex and prior vaccination history recorded. Following vaccination dogs were marked with a temporary collar to distinguish them from unvaccinated dogs and owners were provided with a vaccination certificate. Campaigns ran from 9.00 am to 3.00 pm on a single day at each vaccination station. Since 2017 no additional campaigns have been conducted, except for some localized vaccinations in Lindi Urban and

Kilwa districts in 2019 in response to outbreaks.

**Post-vaccination transects**: From 2013 onwards, following the completion of vaccination campaigns, transects were conducted to record the numbers of vaccinated dogs (marked by temporary collars) and unvaccinated dogs in two randomly selected sub-villages in each of a subset of villages. Transects were completed by LFOs who walked or cycled along transect routes on the evening of the campaign day as detailed in Sambo *et al.* (2018).

**Vaccination coverage**: Data from the post-vaccination transects were used, along with reported numbers of dogs vaccinated during campaigns, to estimate the dog population in each village at the time of each vaccination campaign, using the approach described by Sambo *et al.* (2018). Where transect data were not available for a given village and campaign, population estimates were obtained indirectly, based on transects conducted in the same village but during other campaigns or (if no transects were available for the village) on the overall human/dog ratio for the district, estimated from the projected human population size and a district dog population estimate from all available transects for the district in that vaccination round. Vaccination coverage in each village and campaign (Figure S1) was then estimated by dividing the recorded numbers of dogs vaccinated by the associated dog population estimates. District-level coverage estimates (Table S3) were similarly obtained after summing numbers of dogs vaccinated and dog population estimates over all villages in a district.

**Table S2: Dog vaccination coverage by district.** Coverage achieved by each round of mass dog vaccination campaigns are shown for each district. Values shown are an average of the level achieved across the entire district and do not show the heterogeneity in coverage.

| **District** | **Vaccination coverage (%) by campaign:** | | | | |
| --- | --- | --- | --- | --- | --- |
|  | **1^st^** | **2^nd^** | **3^rd^** | **4^th^** | **5^th^** |
| **Kilwa** | 23.5 | 36.0 | 39.1 | 38.2 | 47 |
| **Lindi Rural** | 23 | 31.4 | 21.9 | 33.9 | 33.2 |
| **Lindi Urban** | 60.1 | 52.5 | 48.9 | 58 | 50.1 |
| **Liwale** | 13.1 | 18.6 | 16.9 | 25.2 | 19.7 |
| **Masasi** | 29.1 | 28.2 | 34.2 | 34.5 | 28.2 |
| **Masasi Township Authority** | 3.3 | 15.4 | 0.8 | 26.5 | 15.8 |
| **Mtwara Rural** | 14.5 | 25.7 | 25.8 | 33.5 | 26.8 |
| **Mtwara Urban** | 34.9 | 24 | 33.7 | 32.7 | 37.6 |
| **Nachingwea** | 34.1 | 47.2 | 54.5 | 54.9 | 46.8 |
| **Nanyumbu** | 21 | 24.8 | 44.3 | 42.4 | 42 |
| **Newala** | 24.2 | 26.7 | 39.2 | 22.1 | 19.8 |
| **Ruangwa** | 27 | 27.5 | 29.8 | 45.4 | 54.8 |
| **Tandahimba** | 22.8 | 23.9 | 31.4 | 35.2 | 32.7 |
| **Mean (standard deviation)** | 25.4 (13.5) | 29.4 (10.5) | 32.3 (14.1) | 37.1 (10.7) | 35 (12.6) |
| **Median (range)** | 23.5  (3.2-60.1) | 26.7  (15.4-52.5) | 33.7  (0.8-54.4) | 34.5  (22.1-58) | 33.2  (15.8-54.8) |


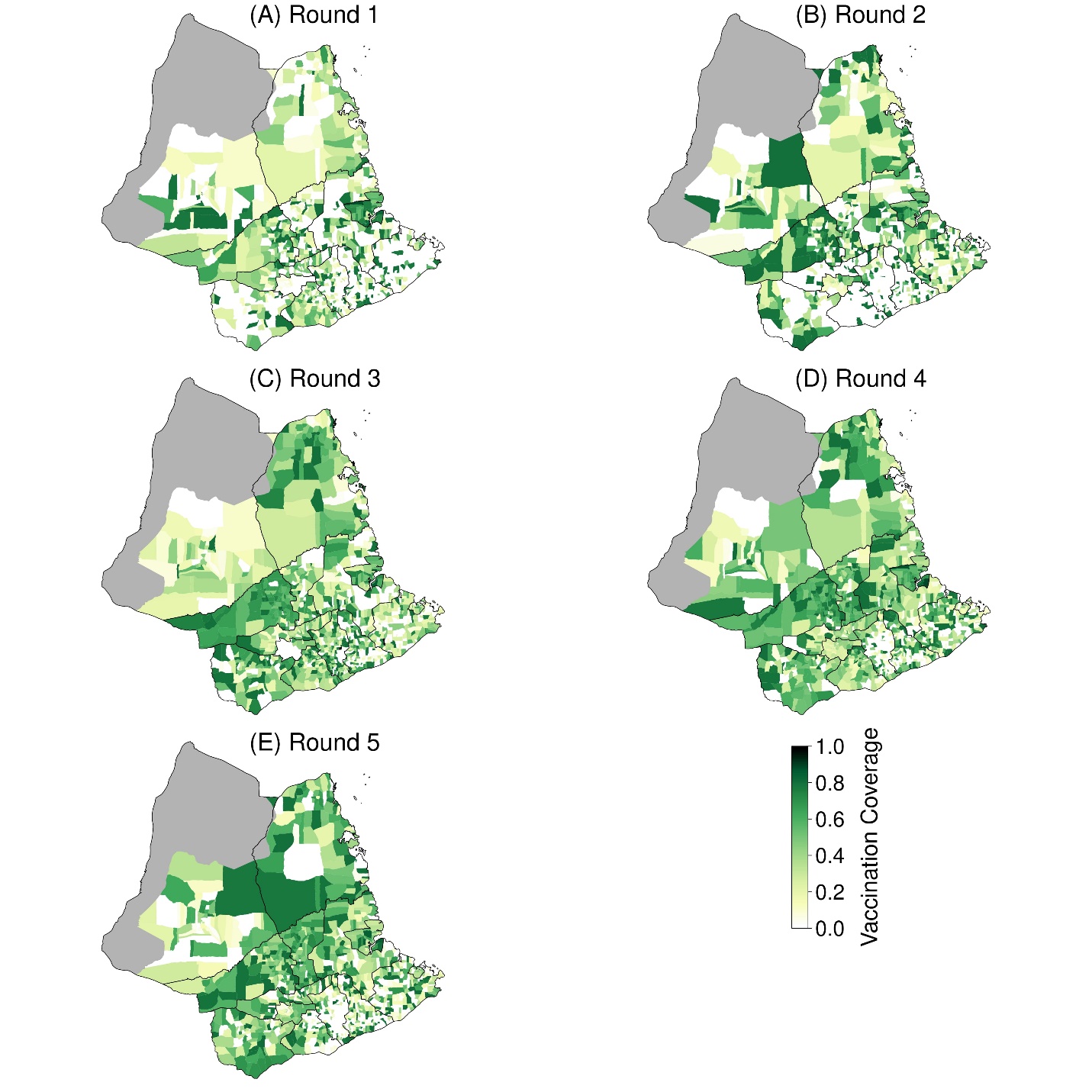


**Figure S1: Estimated vaccination coverage by vaccination round.** Coverage achieved in the southeast Tanzania study area during each vaccination round between 2011 and 2016. The darker the shading, the higher the vaccination coverage.

**Appendix S2: Parameter estimation**

Estimation of the serial interval distribution was carried out using a maximum likelihood based approach, specifically through fitting gamma, Weibull and lognormal distributions to the times between the onset of clinical signs in a primary rabies case and the onset of clinical signs in known secondary cases in the Serengeti data. Model comparison and identification of the best-fitting distribution was carried out using Akaike’s Information Criteria (AIC). The distance kernel was estimated by fitting to the distances between the locations of known primary cases and secondary probable cases that they contacted. Of the 958 pairs of locations of primary cases and their secondary contacts, 301 had a distance of zero recorded. This was due to both primary cases and secondary contacts being from the same household. For these cases interval censoring was applied over a distance of 0-50 metres. Gamma, Weibull and lognormal distributions, as well as two-component mixtures of gamma, Weibull and lognormal distributions were fitted to these distance data to accommodate potential bimodality. Distribution fitting was carried out using maximum likelihood methods and the best-fitting distributions selected using AIC.

**Appendix S3: Sensitivity analyses**

The additional scenarios explored during sensitivity analysis are outlined below.

- Using exact dates of clinical signs onset, without incorporating uncertainty.
- Incorporating uncertainty in recorded dates (0, +/- 7, +/- 14 or +/- 28 days) but allowing progenitors to have a date of onset up to 56 days after the primary case onset. An upper limit of 56 days was chosen to allow for the maximum uncertainty of 28 days recorded in both the primary and secondary case.
- Evaluating only the single most likely progenitor for each case rather than all possible progenitors.
- Using parameters for the distance kernel but with 100 metres as the upper limit for interval censoring instead of 50 metres.
- Using the 95th percentile of the distributions for serial interval and spatial kernel as the cut-off values.
- We posited that dog rabies cases may be better observed than wildlife cases given their proximity to humans. To explore the impact that observation bias might have on inferred species-to-species transmission, analyses were undertaken on subsampled data. Trees were constructed using 60%, 75% or 90% of dog cases but all wildlife and cat (*Felis catus*) cases. For each scenario, sampling with replacement was used to generate a population for transmission tree construction and repeated 10,000 times with mean levels of species-to-species transmission calculated as described in the main text.

**Table S3: Incidence of human rabies exposures.** Mean incidence of exposures to suspected rabid animals per 100,000 people for each district are presented over the course of the study period.

| **District** | **Exposures from all species per 100,000 people** | **Exposure from domestic animals only per 100,000 people** | **Exposures from wildlife only per 100,000 people** |
| --- | --- | --- | --- |
| Kilwa | 6.6 | 6.4 | 0.2 |
| Lindi Rural | 2.2 | 1.7 | 0.5 |
| Lindi Urban | 1.3 | 1.3 | 0 |
| Liwale | 3.9 | 2.7 | 1.1 |
| Masasi | 2.2 | 1.5 | 0.7 |
| Masasi Township Authority | 3.4 | 3.1 | 0.3 |
| Mtwara Rural | 5.4 | 1.4 | 4.0 |
| Mtwara Urban | 0.9 | 0.7 | 0.2 |
| Nachingwea | 5.0 | 2.0 | 2.9 |
| Nanyumbu | 3.6 | 0.9 | 2.7 |
| Newala | 3.0 | 1.7 | 1.3 |
| Ruangwa | 4.3 | 2.4 | 1.9 |
| Tandahimba | 3.7 | 1.6 | 2.0 |

**
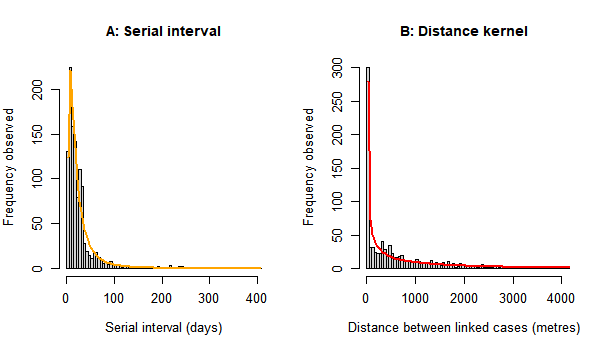
**

**Figure S2: Best-fitting distributions for rabies serial intervals and distance kernels.** Data on serial intervals and distance kernels from contact tracing of rabid domestic dogs in Serengeti District, northern Tanzania were used for parameter estimation. Data are illustrated by the histograms with the best-fitting distributions represented by the overlying lines.(A) observed serial intervals between dog rabies cases with the best-fitting lognormal distribution. (B) observed distances between domestic dog rabies cases with the best-fitting gamma distribution shown. An upper limit of 50m was used for the interval censoring of recorded zero values. The x-axis has been truncated at 4000m to allow easier visualisation of the data. The maximum observed distance was 20713m


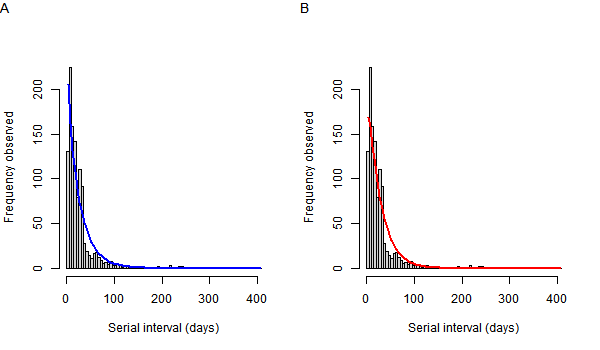


**Figure S3: Alternative distributions for the serial interval.** Data from dogs in Serengeti District, Northern Tanzania are illustrated by the histograms with the fitted distributions overlying. (A) Blue line illustrates the best-fitting Weibull distribution. (B) Red line illustrates the best-fitting gamma distribution.


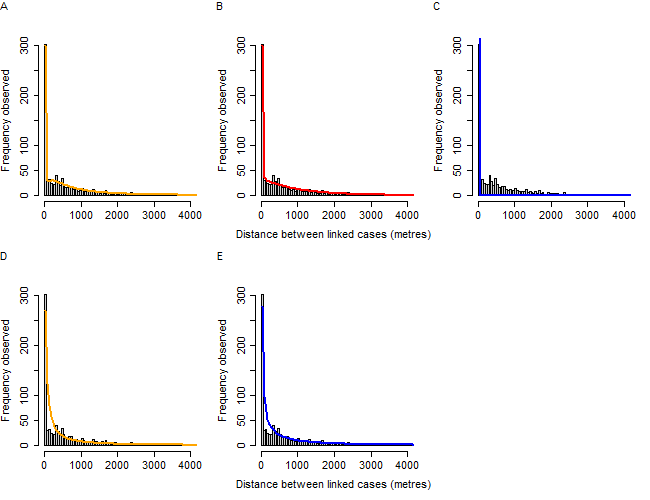


**Figure S4: Distance kernel with fitted distributions using interval censored data at 50m.** The distance between biting animals from Serengeti District, Northern Tanzania are plotted with fitted distributions overlying using an upper limit of 50m for interval censored data. The x axes have been truncated at 4000 metres to allow easier visualisation of the data. The maximum observed distance was 20713m. (A) mixture distribution composed of two lognormal distributions (B) mixture distribution composed of two gamma distributions (C) mixture distributions composed of two Weibull distributions (D) single lognormal distribution (E) single Weibull distribution

**
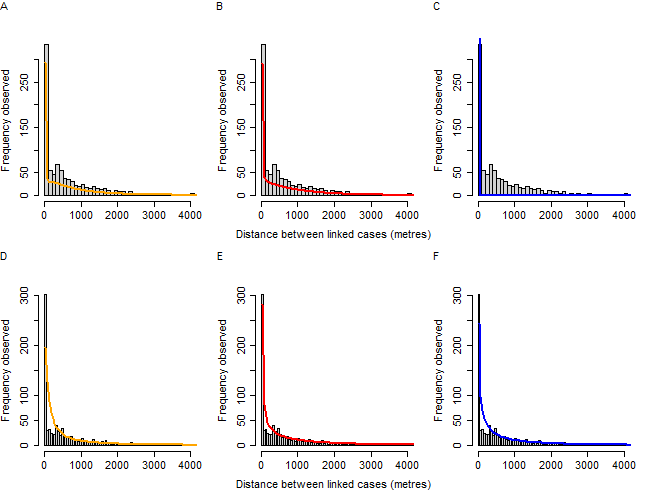
**

**Figure S5: Distance kernel with fitted distributions using interval censored data at 100m.** The distance between biting animals from Serengeti District, Northern Tanzania are plotted with fitted distributions overlying using an upper limit of 100m for interval censored data. The x axes have been truncated at 4000 metres to allow easier visualisation of the data. The maximum observed distance was 20713m. (A) mixture distribution composed of two lognormal distributions (B) mixture distribution composed of two gamma distributions (C) mixture distributions composed of two Weibull distributions (D) single lognormal distribution (E) single gamma distribution (F) single Weibull distribution

**Table S4: Inferred transmissions between species.** The scenario with the lowest cut-off values for serial interval and transmission distance is shown. Cut-off values were generated using the 95^th^ percentile of the serial interval and convolution of two distance kernel distributions and using 100m as the upper limit for interval censoring for transmission distances recorded as zero within the northern Tanzania reference data. These correspond to an upper limit of 80.8 days for serial interval and 5803 metres for transmission distance. Fisher’s exact test values were highly significant, with p-values of less than 0.001 for all of the 1000 contingency tables of inferred transmission.

| **Transmission between species** | **Median number of transmission events**  **(% of overall transmission)** | **Bootstrap 95% confidence interval around median**  **(% of transmissions)** |
| --- | --- | --- |
| Dog - Dog | 77 (45.6) | 65 - 90 (38.5 - 53.3) |
| Dog - Wildlife | 13 (7.7) | 7 - 20 (4.1 - 11.8) |
| Wildlife - Dog | 21 (12.2) | 12 - 29 (7.1 - 17.2) |
| Wildlife - Wildlife | 55 (32.5) | 43 - 67 (25.4 - 39.6) |
| Cat - Dog | 1 (0.6) | 0 - 4 (0.0 - 2.4) |
| Dog - Cat | 0 (0.0) | 0 - 1 (0.0 - 0.6) |
| Cat - Wildlife | 0 (0.0) | 0 - 0 (0.0 - 0.0) |
| Wildlife - Cat | 2 (1.2) | 0 - 5 (0.0 - 3.0) |
| Cat - Cat | 0 (0.0) | 0 - 0 (0.0 - 0.0) |

**Appendix S4: Subsampling of species**

The results from the transmission trees produced by the subsampling analyses are shown in Fig. S7. These results suggest that as the number of dog cases observed decreases, the percentage of inferred transmissions that are dog-to-dog transmissions decreases whilst the percentage that are wildlife-to wildlife increases. However, the 95% confidence intervals are wide and overlap for all scenarios explored.


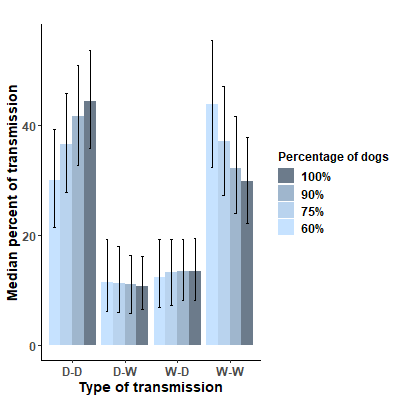


**Figure S6: Subsampled transmission tree analysis.** Results are displayed for the four most common types of transmission. Median percentage of all inferred transmission coloured by the percentage of dogs used within the construction of transmission trees. D-D: Dog-to-dog transmission; D-W: Dog-to-wildlife transmission; W-D: Wildlife-to-dog transmission; W-W: Wildlife-to-wildlife transmission


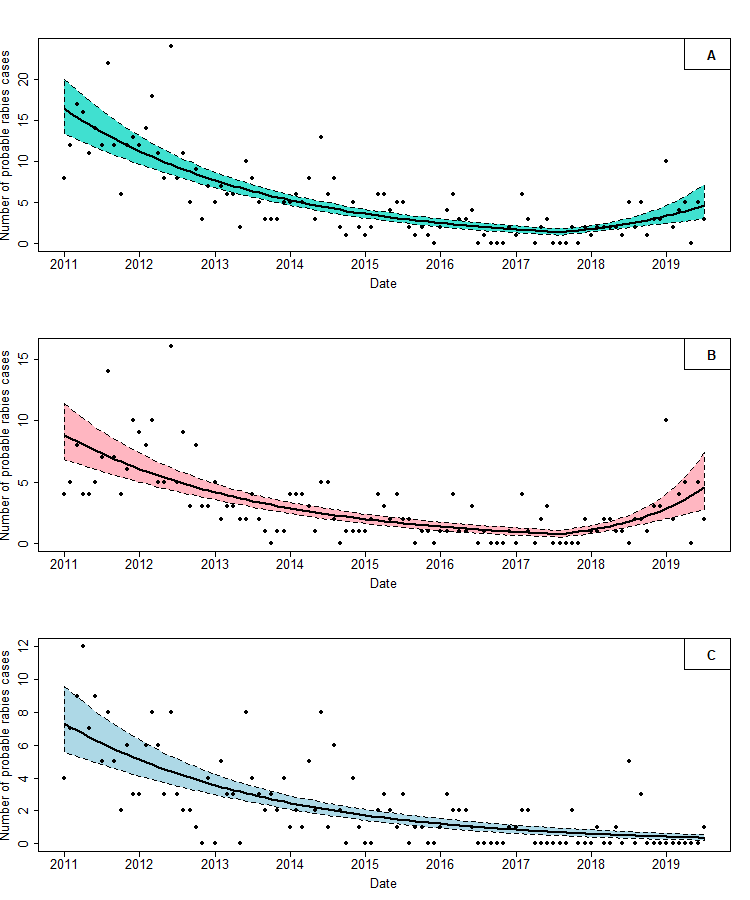


**Figure S7: Regression analysis of probable animal rabies cases per month.** Dots represent the number of probable animal rabies cases for each month. Fitted regression lines from negative binomial regression models are shown in black with the shaded area representing the 95% confidence interval. A) Probable rabies cases in all animal species; B) in domestic animals only and C) in wildlife species only. A statistically significant downward trend in monthly probable rabies cases was found in all three models from January 2011 (p < 0.001, 3.2% (95% CI: 2.7% - 3.7%) reduction per month in all species; p<0.001, 3.2% (95% CI: 2.5% - 3.8% reduction per month in domestic animals only; 3.1% (95% CI: 2.4% - 3.7%) reduction per month in wildlife only). A linear spline was fitted with a knot placed at August 2017 and the change in slopes was found to be significant in the models fitted to cases from all species and to cases in domestic animals only (p < 0.001, 6.3% (95% CI: 3.6% - 9.0%) increase per month in all species; 9.1% (95% CI: 5.8% - 12.4%) increase per month in domestic animals only). For probable cases in wildlife, the slope did not change significantly (p = 0.543) and therefore a single trend was maintained.

**Table S5: Lagged correlation analysis of monthly cases.** Results for monthly cases with lags from 0-11 months are shown. Scenarios with the highest value for the correlation coefficient are highlighted in bold.

| **Lag period (months)** | **Correlation coefficient with domestic dog cases per month leading and jackal cases lagged (p-value)** | **Correlation coefficient with jackal cases per month leading and domestic dog cases lagged**  **(p-value)** |
| --- | --- | --- |
| **0** | **0.525 (<0.001)** | **0.525 (<0.001)** |
| 1 | 0.434 (<0.001) | 0.402 (<0.001) |
| 2 | 0.349 (<0.001) | 0.410 (<0.001) |
| 3 | 0.361 (<0.001) | 0.458 (<0.001) |
| **4** | 0.406 (<0.001) | **0.525 (<0.001)** |
| 5 | 0.256 (0.011) | 0.392 (<0.001) |
| 6 | 0.424 (<0.001) | 0.467 (<0.001) |
| 7 | 0.298 (0.003) | 0.476 (<0.001) |
| 8 | 0.398 (<0.001) | 0.433 (<0.001) |
| 9 | 0.295 (0.004) | 0.509 (<0.001) |
| 10 | 0.432 (<0.001) | 0.512 (<0.001) |
| 11 | 0.262 (0.012) | 0.519 (<0.001) |

**Appendix S5: Logistic regression of cases in relation to population composition**

Sensitivity analyses were undertaken by exploring different scenarios affecting the proportion of the susceptible population composed of domestic dogs or jackals. The scenarios evaluated were:

- Jackal population estimated by assigning them to grid cells with human population density between 0 and 500 per km² at a density of 0.3 jackals per km². Domestic dog vaccination was applied at the median rate of coverage per district to the median estimated domestic dog population.
- Jackal population estimated by assigning them to grid cells with human population density between 1.25 and 500 per km² at a density of 0.3 jackals per km². Domestic dog vaccination was applied at the median rate of coverage per district to the median estimated domestic dog population.
- Jackal population estimated by assigning them to grid cells with human population density between 5 and 500 per km² at a density of 0.3 jackals per km². Domestic dog vaccination was applied at the median rate of coverage per district to the median estimated domestic dog population.
- Jackal population estimates kept constant from the baseline analysis (assigned to grid cells at a density of 0.3 jackals per km² with human population density between 2.5 and 500 per km² at a density of 0.3 jackals per km² ). Domestic dog vaccination was assumed to be zero for the median estimated domestic dog population.
- Jackal population estimates kept constant from the baseline analysis (assigned to grid cells with human population density between 2.5 and 500 per km² at a density of 0.3 jackals per km² ). Domestic dog vaccination was applied at the maximum rate achieved in each district.
- Jackal population estimated by assigning them to grid cells with human population density between 2.5 and 500 per km² at a density of 0.15 jackals per km². Domestic dog vaccination was applied at the median rate of coverage per district to the median estimated domestic dog population.
- Jackal population estimated by assigning them to grid cells with human population density between 2.5 and 500 per km² at a density of 0.5 jackals per km². Domestic dog vaccination was applied at the median rate of coverage per district to the median estimated domestic dog population

All scenarios showed a highly statistically significant (p<0.001) positive association between the proportion of jackals in the susceptible population and the proportion of the total cases occurring in jackals (Fig. S8).


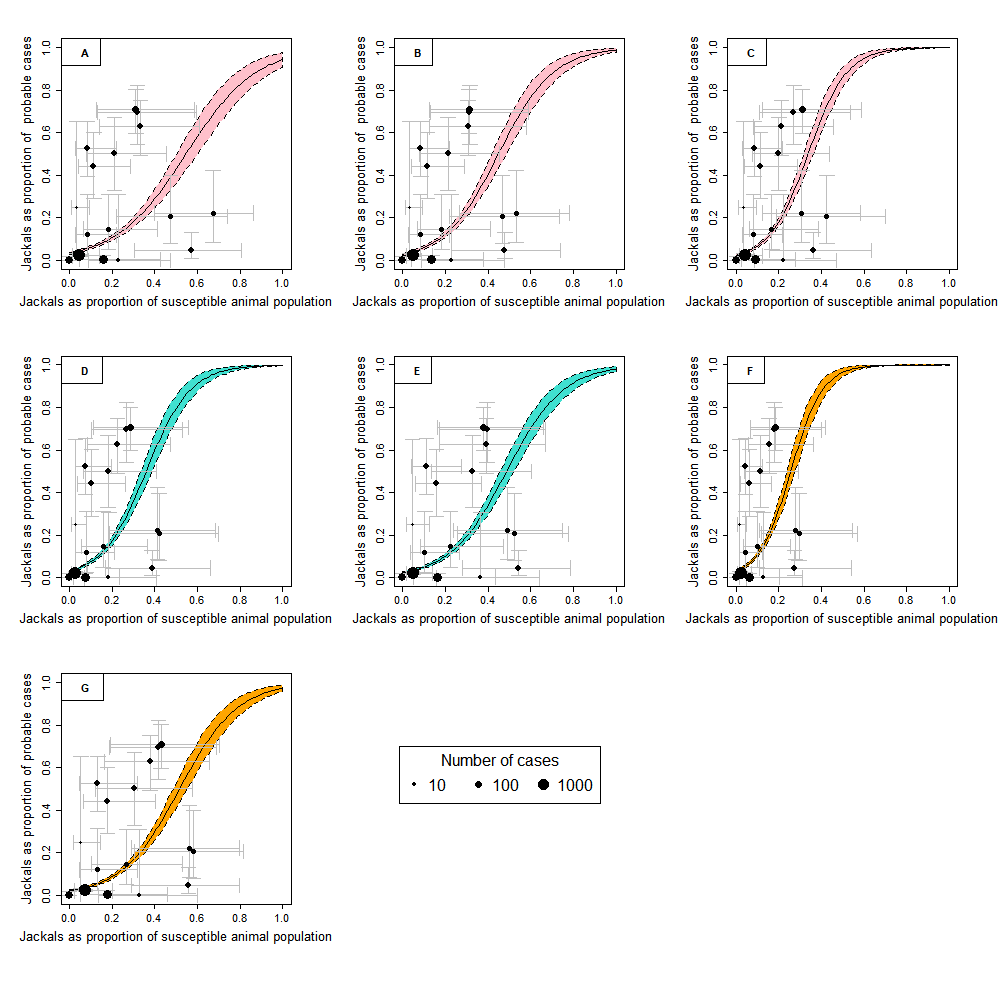


**Figure S8: Jackals as a proportion of the susceptible population versus cases.** The relationship between the proportion of jackals in the susceptible animal population and the proportion of the probable rabies cases observed in jackals was fitted. The susceptible population consists of jackals and unvaccinated dogs assuming A, B, C, F and G) the median level of vaccination coverage achieved in a district; D) zero vaccination coverage and E) the maximum level of dog vaccination coverage. Jackals are applied at a density of 0.3 jackals per km² to areas with between A) 0 and 500 people per km², B) 1.25 and 500 people per km², C) 5 and 500 people per km² and D and E) 2.5 and 500 people per km² and to areas with between 2.5 and 500 people per km² at densities of F) 0.15 jackals per km² and G) 0.50 jackals per km². Probable rabies cases refers to those in jackals and domestic dogs only. Dots represent the 16 districts included in this analysis, scaled by the log10 number of probable cases in that district. Grey bars around the points represent 95% confidence intervals (CIs). The CIs around the proportion of probable cases that occur in jackals are the exact binomial 95% CIs. The CIs around jackals as a proportion of the susceptible animal population was calculated keeping jackal estimates and levels of vaccination coverage constant but incorporating the lower and upper limits of the 95% CIs of the dog number estimates. The fitted logistic regression line is shown in black with the associated 95% CI shown in pink (A, B and C), turquoise (D and E) and orange (F and G).
